# Neurochemistry and functional connectivity in the brain of people with Charles Bonnet syndrome

**DOI:** 10.1177/25158414241280201

**Published:** 2024-10-15

**Authors:** Holly Bridge, Abigail Wyllie, Aaron Kay, Bailey Rand, Lucy Starling, Rebecca S. Millington-Truby, William T. Clarke, Jasleen K. Jolly, I. Betina Ip

**Affiliations:** Wellcome Centre for Integrative Neuroimaging, Nuffield Department of Clinical Neurosciences, Oxford, UK; Wellcome Centre for Integrative Neuroimaging, Nuffield Department of Clinical Neurosciences, Oxford, UK; Wellcome Centre for Integrative Neuroimaging, Nuffield Department of Clinical Neurosciences, Oxford, UK; Wellcome Centre for Integrative Neuroimaging, Nuffield Department of Clinical Neurosciences, Oxford, UK; Wellcome Centre for Integrative Neuroimaging, Nuffield Department of Clinical Neurosciences, Oxford, UK; Wellcome Centre for Integrative Neuroimaging, Nuffield Department of Clinical Neurosciences, Oxford, UK; Wellcome Centre for Integrative Neuroimaging, Nuffield Department of Clinical Neurosciences, Oxford, UK; Wellcome Centre for Integrative Neuroimaging, Nuffield Department of Clinical Neurosciences, Oxford, UK; Vision and Eye Research Institute, Anglia Ruskin University, Cambridge, UK; Wellcome Centre for Integrative Neuroimaging, Nuffield Department of Clinical Neurosciences, Headley Way, Oxford OX3 9DU, UK

**Keywords:** Charles Bonnet syndrome, fMRI, GABA, resting state connectivity, visual hallucination

## Abstract

**Background:**

Charles Bonnet syndrome (CBS) is a condition in which people with vision loss experience complex visual hallucinations. These complex visual hallucinations may be caused by increased excitability in the visual cortex that are present in some people with vision loss but not others.

**Objectives:**

We aimed to evaluate the association between γ-aminobutyric acid (GABA) in the visual cortex and CBS. We also tested the relationship among visually evoked responses, functional connectivity, and CBS.

**Design:**

This is a prospective, case-controlled, cross-sectional observational study.

**Methods:**

We applied 3-Tesla magnetic resonance spectroscopy, as well as task-based and resting state (RS) connectivity functional magnetic resonance imaging in six participants with CBS and six controls without CBS. GABA+ was measured in the early visual cortex (EVC) and in the lateral occipital cortex (LOC). Participants also completed visual acuity and cognitive tests, and the North-East Visual Hallucinations Interview.

**Results:**

The two groups were well-matched for age, gender, visual acuity and cognitive scores. There was no difference in GABA+ levels between groups in the visual cortex. Most participants showed the expected blood oxygenation level dependent (BOLD) activation to images of objects and the phase-scrambled control. Using a fixed effects analysis, we found that BOLD activation was greater in participants with CBS compared to controls. Analysis of RS connectivity with LOC and EVC showed little difference between groups. A fixed effects analysis showed a correlation between the extent of functional connectivity with LOC and hallucination strength.

**Conclusion:**

Overall, our results provide no strong evidence for an association between GABAergic inhibition in the visual cortex and CBS. We only found subtle differences in visual function and connectivity between groups. These findings suggest that the neurochemistry and visual connectivity for people with Charles Bonnet hallucinations are comparable to a sight loss population. Differences between groups may emerge when investigating subtle and transient changes that occur at the time of visual hallucinations.

## Plain language summary

Brain imaging in Charles Bonnet syndrome

In Charles Bonnet Syndrome (CBS), people with sight loss start to see hallucinations that they know are not real but can be disturbing. In this study we used MRI scanning to measure how the chemistry in the visual brain differs in people with CBS and those who have sight loss but no hallucinations. Surprisingly we did not find any differences in chemistry between these groups, suggesting that their brains do not differ greatly. Any differences in how their visual brain works may be too small to measure, or only present when people hallucinate.

## Introduction

Charles Bonnet syndrome (CBS) is a condition defined by the experience of visual hallucinations that accompany a loss of visual function. Over recent years, research into the condition has increased considerably,^1–3^ likely related to recognition that CBS is more prevalent than originally believed. Indeed, a recent meta-analysis suggested prevalence within an age-related macular degeneration (AMD) population to be between 7.2% and 31.6% depending on the type of clinic attended.^3^ There remains significant variability in estimates of CBS prevalence, some of which may be due, at least in part, to the worry of stigma attached to hallucinations.^1^ CBS is not associated with any specific eye disease, and while the condition has been reported mostly in elderly people with vision loss, it can also occur in children with vision loss.^4^ The association with age may therefore be due to the prevalence of eye disease in older age groups and the high number of studies targeting patients with AMD. In terms of the site of vision loss, lesions at any stage of the visual pathway, retinal to central, can be associated with complex hallucinations.^5^ Loss of central vision seems to play a role, with CBS being common in patients with AMD who have impairment in both eyes and loss of central vision.^3^ However, central or significant vision loss is not a requirement.^2^ CBS can occur in patients with glaucoma who have peripheral visual field defects and good visual acuity for example.^4,6–9^ Systematic large-scale prevalence studies are currently lacking that could provide an account of CBS occurrence independent of publication bias, or eye disease prevalence. Overall, the heterogeneity in findings suggests that few clear clinical or demographic markers exist that can reliably indicate who will develop this complex condition. Thus, using vision loss as the primary requirement for CBS, it is estimated that up to 47 million people with moderate vision impairment (vision worse than <6/18) may have the condition.^10^

The pathophysiology of CBS has been included in numerous explanatory frameworks for complex hallucinations (reviewed by Collerton et al.^11^), yet the exact cause of hallucinations remains unknown. Some theories argue that CBS is caused by an interaction of impaired perceptual and attentional processes,^12^ drawing on a generic mechanism that accounts for hallucinations across both neurological and eye diseases. Others suggest that the neural mechanism in CBS is specific to eye disease.^13^ While some of the theories, like the Perception and Attention Deficit model, were successful in accounting for hallucinations in the neurological^14^ as well as the visual domain,^15,16^ the deafferentation hypothesis has proven most parsimonious in the visual domain. The deafferentation hypothesis^17^ states that the loss of visual input decreases cortical inhibition, which leads to spontaneous hyperexcitability in visual areas. Hyperexcitability, brought on by vision loss, may be mediated by changes in presynaptic input,^17^ serotonin and acetylcholine signalling,^5,18,19^ and GABAergic neurotransmission.^20^ Although these studies use different systems to explain CBS, they all involve neural substrates, some of which can be characterised using non-invasive measures in the human brain.

The neural mechanisms underlying CBS are therefore of great importance yet recruiting to magnetic resonance imaging (MRI) studies is challenging because of the increase in MRI contraindications with age. Many patients with CBS are relatively older due to the higher prevalence of age-related macular degeneration compared to other ophthalmic conditions. Given these challenges, many CBS reports involving brain imaging, especially early ones,^21^ are case studies, describing individuals with particularly strong, complex hallucinations.^22^ In many cases, comparable control populations were not scanned,^22,23^ which makes it difficult to dissociate the effects of CBS from the ones due to normal ageing.^24^ For most structural measures, the greatest correlate will be age due to relatively large changes that occur in later life. Thus, while Martial et al. found significant structural differences, the control populations were 40 years younger on average. Indeed, the group study of Firbank et al.,^25^ which had participants carefully matched for age, did not show any structural differences between visually impaired patients with and without CBS.^25^

While there do not appear to be any consistent structural differences in the brain in CBS, what could tell people with CBS apart from those without is atypically high visual activity in specific regions involved in generating hallucinations.^17^ Indeed, a pioneering functional magnetic resonance imaging (fMRI)-study pointed towards a correspondence between the content of hallucinations and activation in functional specialised ventral visual areas in participants with CBS.^26^ A recent electroencephalogram study has shown an increase in neural excitation in the electrophysiological response to visual stimuli in the intact visual field in people with CBS compared to those without.^27^ Within the neural circuitry, over-excitation could be a sign of the visual system rebalancing the lack of external input with stronger internal input through excitatory neurotransmission.^18^ A critical proof of such an underlying mechanism would require the measurement of excitatory and inhibitory neurochemicals, such as glutamate and GABA, in visual areas involved in hallucinations in low-vision patients with and without CBS hallucinations.

Here we compare patients with CBS to an age- and gender-matched group with sight loss but no CBS to determine whether there are any features of the visual system that link to the presence of CBS. We predict that participants with CBS likely show (1) differences in the concentration of inhibitory neurotransmitter GABA and glutamate across the visual cortex; (2) increased resting state connectivity between higher and visual areas, and (3) abnormal activity in the lateral visual cortex to visual stimulation. Our goal is to characterise the visual cortex of participants with CBS by using rigorous non-invasive multi-modal MR imaging techniques.

## Methods

### Participants

The prospective case-controlled, cross-sectional observational study was based at the Wellcome Centre for Integrative Neuroimaging, Nuffield Department of Clinical Neurosciences, University of Oxford. Data collection was performed between August 2021 and July 2023. Our study reporting used guidelines from the Strengthening the Reporting of Observational Studies in Epidemiology (STROBE) checklist for observational studies.

We aimed to recruit two groups of participants: one group with vision loss and CBS and one group with vision loss and without CBS. We did not perform a sample size calculation for this study because no prior study exists that applied magnetic resonance spectrosopy (MRS) in participants with CBS. We recruited 14 participants for the study. Eight had complex visual hallucinations consistent with CBS, and six had sight loss without CBS. Two of the participants with CBS were later withdrawn, one due to ineligibility and the other due to infrequent hallucinations. The final sample size was six participants per group. Participants were recruited from multiple sources, including the Oxford Eye Hospital clinics through the recommendation of an eyecare professional, from mailing lists of the Esme’s Umbrella charity (https://charlesbonnetsyndrome.uk/) and from the public via social media advertisement. Prospective participants were screened in consultation with a research optometrist. Control participants were matched closely to participants with CBS by age, gender, and visual acuity. Participants were recruited for the study if they met all the following inclusion criteria: aged 18–75 years with reduced vision and willing and able to give informed consent for participation in the study. For participants in the CBS group, they also reported experiencing complex Charles Bonnet hallucinations when verbally screened by a researcher using the University of Miami Parkinson’s Disease Hallucinations Questionnaire (UM-PDHQ). Participants could not enter the study if any of the following exclusion criteria applied: pre-existing amblyopia or squint; vision too poor to see any part of the images presented on the MRI-display screen; insufficient understanding of written and verbal English to complete safety screening questionnaires and understand the study requirements; pregnancy or trying to conceive; history of neurological problems; contraindication to MRI. The absence of medical contraindications was confirmed upon screening of the participant’s medical history, including the absence of a history of psychiatric disease. MR spectroscopy-specific contraindications were use of antidepressants or antipsychotic medication.

**Figure 1.**
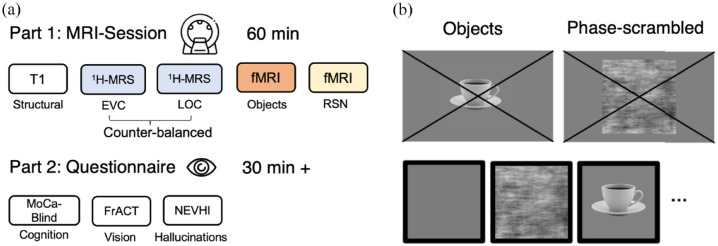
Session design and content. The experimental session included a 60-min MRI session, which was followed by a cognitive test, a visual acuity test and a Hallucination questionnaire. Depending on the extent of hallucinations, the questionnaire session could last 30 min or more (a). In the object-localiser scan, objects were presented with phase-scrambled versions of the same objects (b). MRI, magnetic resonance imaging.

Participants were screened over the phone by a researcher using a series of questionnaires to determine their eligibility for the study. Both cohorts completed an MRI safety screening questionnaire, followed by the impact of visual impairment profile^28^ to measure the impact their visual loss has on their daily lives. Participants for the CBS cohort then also were screened using the UM-PDHQ,^29^ which collected information about their visual hallucinations. If the screening indicated a participant eligible for either the CBS cohort or the control cohort, and safe to go into an MRI scanner, then they were invited to come in for the study Figure 1(a). All questionnaires were conducted by the researcher verbally.

The control participants without CBS were matched for age, gender, and visual acuity to the CBS group (Table 1).

**Table 1.** CBS and controls.

Category	CBS	Control	*p*
Age (years)	52	51	1
Female (%)	33	33	N/A
Binocular VA (LogMAR)	0.38	0.47	0.94
Education (years)	13	16	0.05*
CBS duration (years)	10.71	N/A	N/A
IVI reading	1.69	1.05	0.24
IVI mobility	1.68	1.18	0.48
IVI emotional wellbeing	1.67	1.02	0.06
UM-PDHQ	9.17	N/A	N/A
MoCa-BLIND (/22)	19.5	19	0.29
MoCa-MIS (/15)	12.33	11.83	0.69

*p*: significance value on Mann–Whitney *U* test.

CBS, Charles Bonnet syndrome; IVI, impact of visual impairment profile; MIS, memory index score; MoCa, montreal cognitive assesment test; UM-PDHQ, University of Miami Parkinson’s Disease Hallucinations Questionnaire; VA, binocular visual acuity in LogMAR.

* Statistically significant at alpha = 0.05.

## Magnetic resonance imaging

### Visual stimulation inside the MRI-scanner

Visual stimuli were displayed using Matlab (v.2021b) and PsychToolbox-3 (v.3.0.11). Stimuli were presented using an MR-compatible gamma-linearised LCD screen (BOLDscreen 32; Cambridge Research Systems, Cambridge, UK) positioned at the back of the 3T scanner bore. The screen had a pixel resolution of 1920 × 1200, an aspect ratio of 8:5 and a refresh rate of 60 Hz. The screen was positioned at a viewing distance of 127.5 cm. Participants viewed stimuli presented at the back of the bore through a first-silvered mirror that was fixed to the head-coil at a 45° angle.

### Object localiser task

Functional MRI images were acquired during the presentation of visual stimuli. Stimuli were presented using the Matlab Psychophysics toolbox. A mid-grey image, phase-scrambled objects and intact objects were presented in blocks of 15 s Figure 1(b). Fifteen images of different objects, and their matched phase scrambled counterparts, were presented in each block. Participants were instructed to always keep their eyes on the centre of the display screen. Fixation stability was monitored by the experimenter through an infrared camera positioned at the back of the MR-scanner bore.

### MRI acquisition

MRI data were acquired on a 3-Tesla Siemens Prisma (Siemens Healthineers AG, Erlangen, Germany), using a 64-channel head and neck coil. A high-resolution whole-head T1-weighted isotropic MPRAGE anatomical image (1 × 1 × 1 mm^3^, TR = 2000 ms, TE = 2.03 ms, field-of-view = 256 × 256 mm, 208 slices, flip angle = 8°) was collected for each participant for registration purposes.

### MRS data acquisition

MR spectroscopy data were acquired using a MEGA-PRESS sequence,^30^ consisting of a locally developed version of the CMRR spectroscopy package MEGA-PRESS sequence. MRS acquisition parameters were as follows: voxel size = 20 × 25 × 25 mm^3^; echo time (TE) = 68 ms; repetition time (TR) = 1500 ms; number of spectra = 320 (160 edit-on and 160 edit-off spectra); VAPOR and dual-band editing pulse water suppression; 22.3 ms editing pulse using a 53-Hz bandwidth, which was centred at 1.9 ppm (‘edit-on’ condition) and at 7.5 ppm (‘edit-off’ condition) in alternation; 16-step phase cycling; a total of 8 min 13 s run time. For early visual cortex (EVC) voxel placement, the region of interest was first centred to the occipital midline to cover equivalent portions of the right and left visual cortex, then angled to be parallel to the calcarine sulcus and moved as posterior as possible while avoiding contamination by the cerebellar tentorium and the sagittal sinus. For lateral occipital voxel placement, the voxel was positioned in the right lateral occipital cortex avoiding non-brain tissue and the tentorium. Unsuppressed water references consisting of a single transient were acquired for internal tissue referencing after the main acquisitions for each voxel.

### fMRI data acquisition

A multiband gradient echo sequence was used to acquire fMRI data for the task experiment. Two hundred and ninety volumes were acquired at an isotropic resolution of 2 × 2 × 2 mm^3^ (MB4; TR = 1355 ms; TE = 32.4 ms; field-of-view = 192 × 192; 72 slices; flip angle = 70°). The total scan duration was 402 s. Resting state fMRI data were also acquired using a multiband gradient echo sequence. Six hundred and forty-four volumes were acquired at an isotropic resolution of 2 × 2 × 2 mm^3^ (MB6; TR = 933 ms; TE = 33.4 ms; field-of-view = 192 × 192; 72 slices; flip angle = 60°). A field map was acquired for unwarping of the B0 field.

### Eye movement monitoring inside the MRI scanner

Stable monocular eye position was monitored, but not recorded, using an MR-compatible EyeLink 1000 (SR Research Limited, Ottawa, Ontario, Canada) because participants had low vision and could not reliably detect visual stimuli to calibrate the eye tracker. Researchers confirmed that all participants maintained good fixation during visual stimulation runs.

### MRS analysis

Data analyses were performed using FSL-MRS (v.2.1.17),^31^ part of the open-source FSL toolbox. First, MRS data were converted from TWIX to NIfTI format using spec2nii.^32^ Then, pre-processing was performed using fsl_mrs_preproc_edit for edited MRS data. It included the following steps: coil-combination, windowed averaging of phase and frequency alignment between repeats, eddy current correction, truncation of the FID to remove three time-domain points before the echo centre, removal of residual water peak using Hankel Lanczos singular value decomposition over 4.5–4.8 ppm, phase and frequency alignment between averaged edit-on and edit-off spectra using spectral registration on the 2.5- to 3.5-ppm range. The processing also outputs a phase corrected non-water suppressed reference. The model fitting of the SVS data was implemented using a Linear Combination model as described in Clarke et al.^31^ In essence, basis spectra are fitted to the complex-valued spectrum in the frequency domain by scaling, shifting and broadening them. Basis spectra were grouped into two metabolite groups, with macromolecular peaks allowed to broaden and shift independently of other metabolites. The model fitting was done using the truncated Newton algorithm as implemented in Scipy, and no baseline was modelled. To estimate metabolites in the edit-on minus edit-off difference spectrum, a simulated basis set containing the model spectra for N-acetylaspartate (NAA), N-acetylaspartateglutamate (NAAG), γ-aminobutyric acid (GABA), glutamine (Gln), glutamate (Glu), glutathione (GSH), macromolecules (MM) and combined NAA + NAAG, Glu + Gln + GSH, GABA + sysMM was used (https://git.fmrib.ox.ac.uk/wclarke/win-mrs-basis-sets). The internal reference limits were 1.8–2.2 ppm. As the GABA signal at 3.0 ppm contains co-edited macromolecule signals, as well as homocarnosine,^33^ the signal is referred to as GABA+ macromolecules (GABA+). Metabolites were reported in tissue fraction and tissue relaxation corrected absolute concentration in millimolar per kilogram (mmol/kg). MRS shim quality was measured with the full-width-half-maximum of the inverted NAA singlet in the difference spectrum. MRS voxel positions were reconstructed using FSL FAST called within FSL-MRS. FAST subdivided the high-resolution anatomical image into white matter, grey matter and cerebrospinal fluid and calculated tissue fractions within EVC and LOC voxels.

### fMRI analysis

Pre-processing and all statistical analyses were carried out using FSL tools (v.6.00 www.fsl.fmrib.ox.ac.uk). Pre-processing involved (i) brain extraction using BET, (ii) motion correction with MCFLIRT, (iii) B0 distortion correction using the field map, (iv) spatial smoothing using a 5-mm Gaussian kernel and (v) high-pass temporal filtering. FLIRT^34,35^ was used to register echo planar imaging (EPI) images to the individual structural images (boundary based registration [BBR] method) and FNIRT^36^ was used to register to standard space.

Using FEAT, object blocks and phase-scrambled blocks were included as explanatory variables in a general linear model and contrasted both to fixation and each other. A higher-level mixed effects analysis was performed to determine the mean group response to each stimulus and the contrast between groups. A second fixed effects analysis was also performed to investigate the differences between the two groups, given the small sample size, which is below the recommended size for mixed effects analyses. For all group analyses, age was included as a nuisance regressor.

A region of interest (ROI) analysis was performed to quantify the percentage BOLD signal change to the object and phase stimuli in visual areas V1 and LOC. The V1 mask was taken from the Human Connectome Project atlas^37^ and LOC was from the Harvard-Oxford cortical atlas, implemented in FSL.

### RSN analysis

Resting-state data were pre-processed using FEAT. The initial stage included (i) brain extraction using BET, (ii) motion correction with MCFLIRT, (iii) B0 distortion correction using the field map, (iv) spatial smoothing using a 5-mm Gaussian kernel. No temporal filtering was applied at this stage. FLIRT was used to register EPI images to the individual structural images (BBR method), and FNIRT was used to register to standard space. The second stage used ICA-AROMA non-aggressive denoising^38^ to remove motion-related noise from the pre-processed data. This approach has been shown to be adequate in a patient cohort.^39^ High-pass temporal filtering at 100 s was performed as the final pre-processing stage. Finally, the timeseries were extracted from the cerebrospinal fluid and white matter and used as nuisance regressors. The same ROIs (LOC right and left; V1 bilateral) were used as for the fMRI analysis, but with the two hemispheres considered separately. The seed-based analysis involved extracting the mean timeseries from the pre-processed data across voxels for each ROI and using it as an explanatory variable for a FEAT analysis using a general linear model.

To determine whether the extent of functional connectivity with LOC and V1 was related to the extent of hallucinations, a higher-level fixed effects correlation analysis was performed across all 12 participants. In each case, the right ROI was used since the LOC neurochemical concentration was measured in that hemisphere. However, the results were similar using both ROIs. Since a fixed effects analysis was used, the results were thresholded at *z* > 8 to consider only the regions with the highest correlations.

### NEVHI questionnaire and analysis for generating predictors for MRI-RSN and MRS

We used the North-East Visual Hallucinations Interview (NEVHI) for participants to report their visual experiences.^40,41^ If a hallucination lasted for ‘seconds’, this was quantified as 0.1 min. Hallucinations lasting minutes were assigned the value of 5 min. Reports of ‘minutes/hours’ were assigned 60 min, and ‘hours’ were assigned 120 min. The final hallucination score was generated by calculating the duration × frequency for each hallucination type and then averaging across all hallucination types for each participant to give the typical hallucination minutes per month for each participant. This score was finally multiplied by the distress experienced to provide a final severity score that accounted for the frequency of hallucinations and the distress caused by them. The higher the score, the worse the hallucinations. NEVHI scores were used as a correlate for metabolite concentrations.

### Definition of visual acuity

Visual acuity (VA) is representative of high contrast central vision and is the most commonly used measure in ophthalmology for monitoring of disease.^42^ We used the Freiburg Acuity Test (FrACT) (https://michaelbach.de/fract/) for the measurement of VA as it can measure a wider range of vision than traditional charts and is therefore better suited for low vision.^43^ FrACT measurements were conducted using a 27-inch DELL U2713HM monitor (pixel resolution = 2560 × 1440). This setup allowed measurement to a maximum of 2.90 LogMAR. Participants were sat as near to 2 m from the monitor as possible, then brought forward as needed in low vision. Exact distances from the eye to the screen were measured using a tape measure and entered in the settings allowing the FrACT software to calculate dimensions for each participant to ensure accurate results. The task was completed with the room lights switched off to ensure stable illumination. Participants were instructed to read the letters on the monitor while covering their right eye, then while covering their left eye and then while using both eyes. Participants with visual correction were instructed to wear this during the task, and all participants were told not to lean forward in their seats, to avoid changing the distance measurements.

### Montreal cognitive assessment version for the blind

Cognitive assessment of each participant was assessed using the Montreal cognitive assessment version for the blind (MoCa-BLIND) questionnaire.^44^ All examiners were formally accredited to undertake this assessment, a validated standard instrument for cognitive assessment. Scores of 18 or less are considered to indicate some degree of cognitive impairment.

### Statistical analysis

The main statistical comparisons were between CBS and age- and gender-matched control groups. Neurochemical outcome measures were MRS-derived neurometabolites (GABA+, Glx) from the two regions of interest (EVC, LOC). Neural activity outcome measures were fMRI responses to visual stimuli and resting-state connectivity maps seeded from the EVC and LOC. MRS metabolite measures were correlated with NEVHI scores.

Data analysis was performed using RStudio (RStudio Version 2023.06.0+421 (2023. 06.0+421), the pandas software library (v.2.0.3) using Python^45^ and plotted using Jupyter Notebook. All data were tested for assumptions of normality using the Shapiro Wilk test, with *p* > 0.05 considered normally distributed (*stats.shapiro*). If data were not normally distributed, equivalent non-parametric tests were used. Equality of variance was assessed using the Levene’s Test of means (*scipy.stats, levene*), with *p* > 0.05 considered equal variance. *P*-values were greater than 0.05, showing that variance between groups was not significantly different.

A linear mixed model analysis with the fixed effects of ‘group’ and ‘visual area’ was used to assess the effect of stimulation condition on MRS shim quality while modelling random effects of ‘age’ (*lme4*). The variable ‘sex’ was added as a fixed effect dummy variable. We used *lmerTest* to obtain *p*-values directly from the lmer function. As there were no interactions between fixed effects *(lmer(dv* *~* group*visual area*sex *+* *(1|age), data=dat))*, we used Type II Analysis of Variance Table with Kenward–Roger’s method to test for significance of each fixed effect individually. The model syntax was *lmer(dv* *~* *group* *+* *visual area* *+* *sex* *+* *(1|age), data=dat)*. A two-sided Mann–Whitney *U* test (*scipy.stats.mannwhitneyu*) was used to test for differences in demographic and questionnaire data between groups.

A sensitivity power analysis using G*Power^46^ showed that a two-tailed independent means measures *t* test with six participants would be sensitive to large sized effect (Cohen’s *d* = 1.8) with 80% power (alpha = 0.05). This means that the analysis can reliably detect effects at or above Cohen’s *d* = 1.8.

## Results

All participants took part in the questionnaire and visual acuity (VA) measurements. While participants with and without CBS had matched binocular visual acuity (Table 1, VA), only participants with CBS had non-zero scores for the NEVHI Hallucination score. Hallucination scores for each participant are shown in Table 2.

**Table 2.** Participant demographics and test scores.

Group	sub	Age/Sex	Match	Diagnosis	MoCa	VA	NEVHI	Duration (years)
CBS	sub-001	57/F	sub-014	Rod/cone and macular dystrophy	21	1.19	1350	4.5
	sub-002	33/M	sub-006	Left eye retinal detachment	21	0.54	5	25.5
	sub-003	35/F	sub-012	Binasal hemianopia	17	−0.19	5760	6.5
	sub-005	70/M	sub-011	Retinitis pigmentosa	19	0.3	13	10.5
	sub-007	54/M	sub-013	Hereditary retinal dystrophy	19	0.34	3897.5	14.75
	sub-009	60/M	sub-008	Stargardt disease	20	0.1	3600.08	2.5
Control	sub-006	39/M	sub-002	Optic atrophy	19	0.95	0	
	sub-008	65/M	sub-009	Inherited macular degeneration	19	−0.07	0	
	sub-011	70/M	sub-005	Macular dystrophy	19	0.88	0	
	sub-012	35/F	sub-003	Stargardt disease	19	0.93	0	
	sub-013	47/M	sub-007	Retinitis pigmentosa	19	−0.13	0	
	sub-014	52/F	sub-001	Retinitis pigmentosa	19	0.28	0	

CBS, Charles Bonnet syndrome; F, female; M, male; MoCa, montral cognitive assessment test; NEVHI, North-East Visual Hallucinations Interview; VA, binocular visual acuity in LogMAR.

**Figure 2.**
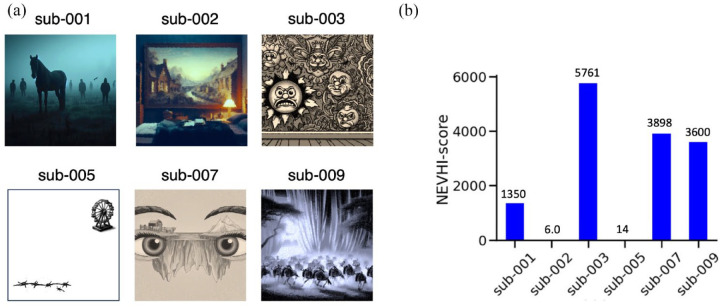
CBS visual hallucinations. Participants with CBS described unique complex hallucinations during the NEVHI questionnaire. (a) Visual experiences included: a horse and people standing around (sub-001); a room in the childhood house, long gone (sub-002); cartoon faces and Victorian wallpaper (sub-003); a miniature Ferris wheel and barbed wire (sub-005); a cattle farm in one eye and an iceberg in the other eye (sub-007) and wildebeests running silently across the blinds at night, like a black-and-white film (sub-009). Images were generated using a generative artificial intelligence app Dall-E 3. The distress felt by participants during their hallucinations contributed to the quantification of the NEVHI-scores (b), a metric derived from the questionnaire reflecting the frequency, duration and reported distress of hallucinations. CBS, Charles Bonnet syndrome; NEVHI, North-East Visual Hallucinations Interview.

Participants with CBS reported seeing a mixture of simple to complex hallucinations (Figure 2(a)). None of the participants with CBS reported hearing sounds in addition to their visual hallucinations, and none thought that their hallucinations were real. Participants with CBS displayed a range of scores, showing that there is heterogeneity in the frequency and duration of hallucinations (Figure 2(b)).

### No evidence for altered neurochemistry in CBS compared to controls

**Figure 3.**
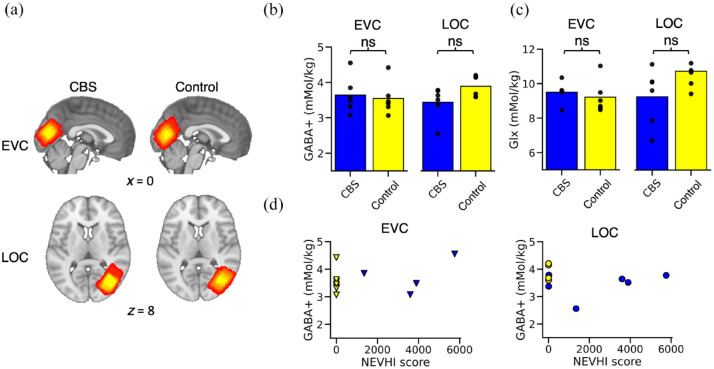
MRS results. Group MRS voxel position of the EVC voxel displayed on the MNI-152 standard brain template for participants with CBS (a, left, top) and controls (a, right, top). The position of the LOC in the right hemisphere for CBS (a, left, bottom) and controls (a, right, bottom). MNI-152 *x*- and *z*-coordinates indicate slice position in mm. Mean concentrations of GABA+ (b) in the EVC and LOC for CBS (blue) and controls (yellow) and of Glx (c) in the EVC and LOC for CBS (blue) and controls (yellow). (d) Scatterplot shows the relationship between GABA+ and the NEVHI-score in CBS (blue markers) and controls (yellow markers) for the EVC and for the LOC. Dots show individual participants. CBS, Charles Bonnet syndrome; EVC, early visual cortex; LOC, lateral occipital cortex; NEVHI, North-East Visual Hallucinations Interview; ns, not statistically significant.

The aim of the analysis was to compare concentrations of the inhibitory neurotransmitter GABA in the lateral occipital cortex in participants with vision loss with (‘CBS’) and without CBS hallucinations (‘controls’). Due to the presence of complex hallucinations in CBS, we hypothesised that GABA+ levels in the ventral stream area LOC, which is related to visual object processing, would reflect differences between CBS and controls, whereas GABA+ in the EVC would not. Six participants were included in each group. We used a linear mixed model to test whether group or region-of-interest (EVC or LOC, Figure 3(a)) affected the MRS data acquisition quality proxy NAA line width. We found a significant effect of ROI on MRI acquisition quality (*F*(1,12.47) = 5.09, *p* = 0.04), meaning that where the MRS data were measured had an impact on the MRS shim quality. Hence data from the two voxels could not be directly compared. However, there was no significant effect of group on MRS quality (*F*(1,18.42) = 0.10, *p* = 0.75), meaning that metabolite values could be compared across CBS and controls within ROI.

The GABA+ concentration (Figure 3(b)) in the EVC for participants with CBS was 3.64 ± 0.51 mmol/kg (mean ± SD), and for controls was 3.55 ± 0.47 mmol/kg. GABA+ data were normally distributed, and there was no difference in EVC GABA+ between CBS and controls (*t*(10) = 0.33, *p* = 0.74). The mean GABA+ concentration in the LOC for CBS was 3.44 ± 0.46 mmol/kg and for controls was 3.90 ± 0.30 mmol/kg. Values were not normally distributed, and a Mann–Whitney *U* test found no significant difference between the LOC GABA+ concentration of participants with CBS and controls; *U* = 8, *p* = 0.13. Hence we did not find significantly different GABA+ in this object selective region as originally hypothesised. We also found no significant difference between the means of CBS and control groups for the glutamate and glutamine complex Glx (Figure 3(c)) in the EVC (*t*(10) = 0.62, *p* = 0.55 or the LOC (*t*(10) = −1.88, *p* = 0.09).

Next, we investigated the relationship in patients with CBS between their Hallucination score and GABA+ concentrations in both EVC and LOC voxels (Figure 3(d)). Hallucination scores were not normally distributed, and we calculated a Spearman’s Rank correlation to assess the relationship between Hallucination scores and GABA+ in the EVC or LOC. There were no significant correlations between GABA+ and Hallucination scores for EVC (*r*(4) = 0.26, *p* = 0.62) or the LOC GABA+ (*r*(4) = 0.26, *p* = 0.62). Data from controls are plotted alongside the participants with CBS but were not included in the correlation analysis. In summary, our MRS results showed no evidence supporting a link between GABAergic inhibition in area LOC and the intensity of visual hallucinations in the participants with CBS.

In this section, we quantified neurochemical concentrations non-invasively in two locations in the visual cortex in participants with CBS and controls, the EVC, and the LOC. Overall, our results suggest that patients with CBS did not have a significantly altered neurochemistry in the visual cortex relative to control participants with similar gender, age, and visual acuity loss.

### CBS and control participants showed fMRI responses to both images of objects and phase-scrambled images

**Figure 4.**
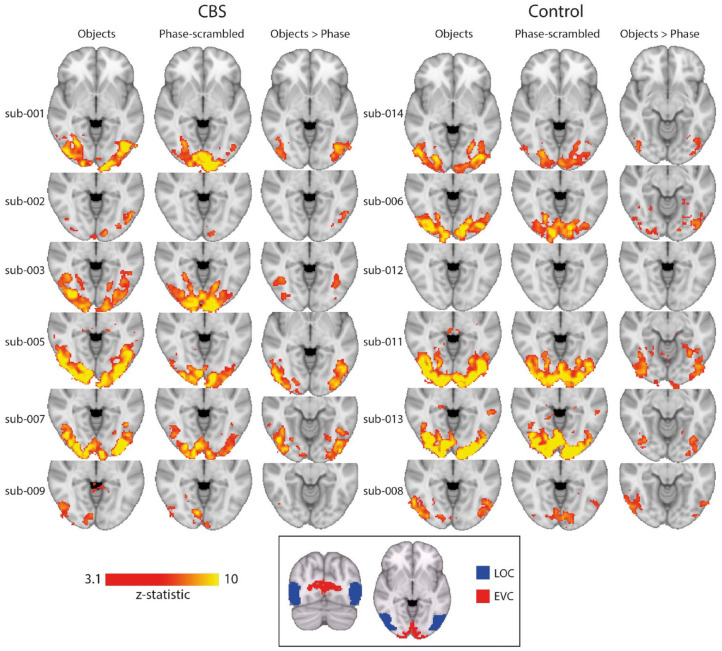
fMRI responses to images of objects and phase-scrambled versions of the objects, alongside the regions more responsive to objects compared to the phase-scrambled images. Responses are shown for participants with CBS (left side) and their matched control participant (right side). Box shows the location of areas lateral occipital cortex (LOC) and early visual cortex (EVC). CBS, Charles Bonnet syndrome.

To determine whether visually evoked activity was abnormal in people with CBS, we next used fMRI to investigate the neural responses to images of objects, and images of the same objects that were phase-scrambled. All participants with CBS and 5/6 visually impaired participants without CBS showed significant activation to images of both objects and phase-scrambled versions of the same objects. Figure 4 shows the activity patterns evoked by the objects and phase-scrambled images in participants with CBS (left side) and the matched controls (right side). The regions that were more responsive to objects compared to the phase-scrambled images (Objects > Phase) are also shown.

**Figure 5.**
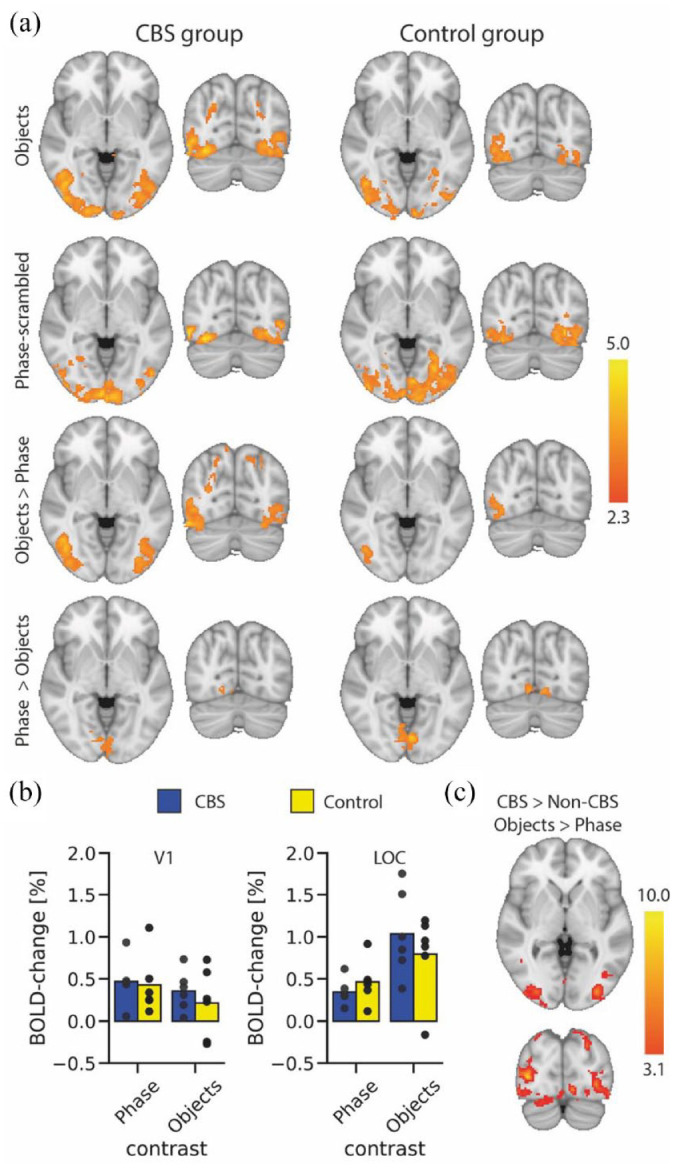
BOLD-fMRI responses to visual stimuli. (a) Group responses to viewing of objects (top row) shows extensive activation in lateral visual regions in the CBS group and slightly lower responses in the control group. In contrast, activity to the phase-scrambled images (second row) appears more extensive in the control group. The contrast of activation to objects compared to phase-scrambled images shows bilateral activation in the lateral visual cortex for the CBS group, but only unilateral in the control group. Both groups show more activation in medial visual areas to the phase-scrambled images compared to the objects. The region-of-interest analysis (b) shows no difference in %BOLD signal change between the two groups either in EVC or LOC. However, there is a hint in LOC that participants with CBS show greater activation to objects and lower activation to phase scrambled stimuli. This difference is reflected in the fixed effects (c) results showing greater activation in LOC in participants with CBS to objects compared to phase-scrambled stimuli. Scalebar in each case shows the *z*-statistic of the activation. CBS, Charles Bonnet syndrome; EVC, early visual cortex; LOC, lateral occipital cortex.

To determine whether there was any difference in the response levels between the two groups, a mixed effects group-level analysis was performed. Both CBS and control groups showed greater activation in early visual areas to the phase-scrambled stimulus (Figure 5(a)). In contrast, the CBS group appears to show more extensive activity than the control group to the images of objects. However, this was not reflected in the direct comparison between the groups, likely due to the small number of participants and the use of a mixed effects analysis which is conservative. To explore the data further, we performed a fixed effects analysis to investigate group-level differences too weak to be present in the mixed effects analysis, and which are only valid for the current populations. Figure 5(c) shows the results of the fixed effects analysis for the difference between the object and phase stimulus. The participants with CBS show more activity in area LOC than the patients without CBS.

Since we predicted that the differences in activation would likely be in visual LOC, we also performed a region-of-interest analysis for visual areas EVC and LOC (Figure 5(b)). An ANOVA showed that there was no effect of patient group in either ROI (EVC: *F*(1, 10) = 0.5; LOC: *F*(1,10) = 0.09). There was, however, a significant effect of stimulus type in LOC (*F*(1,10) = 29.0; *p* = 0.0003) but not EVC (*F*(1,10) = 1.4)). There was no significant interaction in either ROI (EVC: *F*(1, 10) = 0.1; LOC: *F*(1,10) = 0.4).

### Subtle differences in functional connectivity between participants with and without CBS

**Figure 6.**
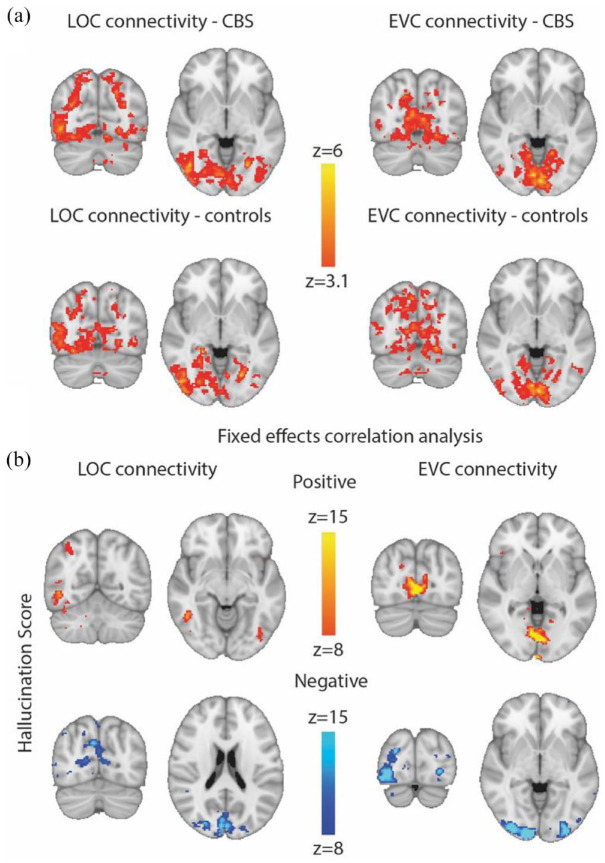
Resting state connectivity with visual areas. (a) Shows the results of the mixed effects group analysis indicating the regions of the occipital lobe that are significantly correlated with the time series in LOC and EVC. The pattern of areas is similar in participants with and without CBS for both LOC and EVC. (b) Shows the occipital regions that show a significant correlation with Hallucination score using a fixed effect analysis (and therefore only valid for the current population). For the correlation with right LOC, there is a significantly greater connectivity within ventral LOC in those with the strongest hallucinations. In contrast, the bottom row shows that those with the strongest hallucinations have relatively low correlation in medial parietal and anterior calcarine cortex. For EVC there is greater correlation within EVC for those with stronger hallucinations, but weaker correlation outside of EVC (bottom row). CBS, Charles Bonnet syndrome; EVC, early visual cortex; LOC, lateral occipital cortex.

A seed-based analysis of the resting data was performed to identify the regions of the brain that are significantly correlated with the two regions of interest, EVC and LOC. The network of areas with the highest correlation to right LOC and bilateral EVC are shown in Figure 6(a). Note that the connectivity patterns of left and right LOC were similar, so the right side is shown as that is the hemisphere where the MRS voxel was located. There was very little difference between the correlation maps for the groups with and without hallucinations for both LOC and EVC. The connectivity map from the right LOC includes the intraparietal sulcus and looks similar to the fMRI activation pattern to the presentation of objects shown in Figure 5. There is a slight lateralisation to the right hemisphere due to the use of the right LOC time series. The connectivity pattern of bilateral EVC is more medial, again reflecting the fMRI response to phase-scrambled images compared to the objects (Figure 5).

There was a considerable amount of variability in the strength of connectivity between different individuals but, in addition, even within the CBS group, the Hallucination scores differed considerably. We therefore performed a correlation analysis to determine the brain areas that showed a relationship between Hallucination score and connectivity with LOC and EVC. Figure 6(b) shows the regions showing either a positive or negative correlation with the connectivity of LOC and EVC. On the left, there is a positive correlation between connectivity within LOC and Hallucination score. Similarly, EVC shows a positive correlation with the Hallucination score. This suggests that both EVC and LOC have a stronger local correlation in participants with stronger hallucinations. In contrast, the lower row shows the regions that have greater correlation in those without hallucinations. For LOC this is the medial parietal and anterior calcarine sulcus, and for EVC it was likely visual areas V2 and V3.

There were very few areas that correlated positively with Hallucination score, but Figure 6(b) shows that for both LOC and EVC, the correlation pattern of regions in the occipital lobe was anti-correlated with the Hallucination score. This indicates that, contrary to our hypothesis, correlation with both LOC and EVC is greater within the occipital lobe in people with fewer hallucinations.

## Discussion

In this study we investigated the neurochemistry, visually evoked neural responses and resting state connectivity in people with visual impairment with and without CBS. The principal aim was to establish whether there were any neural markers for increased excitability that could explain why some individuals experience hallucinations and others do not. Our paper demonstrated that across all the MRI-measures considered, there were no consistent differences between those participants with and without CBS. The evidence from our multi-modal neural characterisation suggests that the cortical substrates of Charles Bonnet participants are broadly within normal limits for a sight loss population.

### No difference in neurochemical concentration of GABA+ or Glx in participants with CBS

The major hypothesis for this study was that the complex hallucinations experienced by participants with CBS would be linked to a difference in either inhibitory (GABA+) or excitatory (Glx) neurotransmitter concentration. The logic behind these hypotheses was that lower GABA+ in area LOC might lead to increased activity in this object-related visual area compared to those without hallucinations. An increase in Glx would have a similar effect on the ratio of excitation and inhibition. There was, however, no significant difference in the concentration of GABA+ or Glx in either area LOC or EVC in our small but carefully matched group of participants, nor was there a correlation with the Hallucination score. These results show that visual cortex neurochemistry is comparable to those who experience vision loss but no hallucinations. What could explain this result?

As our Hallucination questionnaire revealed, no participant experienced the same images as another (Figure 2). Although vivid, the visual hallucinations are transient events with a high interindividual variability in frequency and content. This suggests that the time course of the events may be key to detecting states in which hallucinations occur, on an otherwise structurally typical visual cortex. In support of this idea, an elegant study by Hahamy et al. showed comparable patterns of fMRI response between participants with CBS during visual hallucinations and control participants who imagined the content of the hallucinations.^47^ The only difference between the two groups was that participants with CBS demonstrated a slow buildup of activity across the visual cortex prior to the hallucinations; activity that was not present in the matched controls. In the current study, GABA+ and Glx were averaged over time resulting in no differences between participants with CBS and controls in either EVC or LOC. Future studies could attempt to track the neurochemistry before and during hallucinations to time-lock the neurochemistry to spontaneous activity in the visual cortex.

It is also the case that we only considered GABA+ and Glx in this study, the major inhibitory and excitatory neurotransmitters in the brain that are also MR visible. We cannot rule out the involvement of other neurotransmitters in CBS hallucinations, such as acetylcholine which is not directly detectable using MR spectroscopy. In addition, due to time limitations, we only measured the neurochemistry in area LOC and EVC. Since the content of hallucinations in some cases may be linked to emotional state, there may be other brain areas, including the limbic system, that show altered neurochemistry. The emotional impact of the hallucinations may be less variable than the visual content, but this requires further investigation.

### Considerable inter-individual variability in hallucination content and strength

In CBS, the nature of hallucination varies considerably between individuals, with hallucinations often divided into ‘simple’ and ‘complex’.^48^ For the current study, we limited our inclusion criterion to those experiencing complex hallucinations, that is those made up of complicated objects, people, animals or scenes. Potential participants who only experienced simple hallucinations, such as patterns of lines or spots, were excluded.^19^ Even with this restriction, Figure 2 shows the range of hallucinations experienced. In each case the participants were able to clearly describe what they experienced. In addition to the content, the duration and frequency of the hallucinations were both variable, ranging from a duration of a few minutes, and occurring weekly, to almost continuous. The extent of differences even in this small population is likely to reflect the wider population of people with CBS.^1,49^

### Evoked activity to images of objects shows little difference across CBS and non-CBS control groups

Since our hypothesis was that processing in object-sensitive area LOC is likely to be abnormal, we chose to measure the neural response to images of objects and scrambled objects and compare the response between the two groups of participants. Even though all participants had visual impairment, almost all showed some evoked activity. Those with Stargardt disease, both with and without CBS, showed the least activity, reflecting the loss of central vision. Both groups showed the expected pattern of activity, with area LOC significantly more active for the images of objects compared to scrambled objects, and V1 more active to the scrambled image stimuli. While the conservative mixed effects analysis did not reveal any differences between the two participant groups, the fixed effects analysis that is valid for this population only showed a greater difference between activation to the objects and phase-scrambled objects in the participants with CBS than those without. This was also hinted by the region-of-interest analysis that showed greater activity in LOC to objects and lower activity to phase-scrambled objects in participants with CBS compared to non-CBS.

There is no simple relationship between the BOLD signal measured with fMRI and neuronal excitability, but it may be that the lateral visual areas of the brain in people with CBS are more sensitive to visual processing of objects than those without.

### Even resting state correlations do not differ between CBS and non-CBS control groups

The specific reason for investigating correlations at rest is that there is no confound with the extent of visual function, as might be the case when presenting visual stimuli. As an additional measure of brain function, it allowed us to scope any differences in connectivity between the visual cortex and higher-level control areas that involve attentional deployment. However, the two groups of participants showed very similar patterns of connectivity at rest, providing no support for models arguing for abnormal interactions between visual and cognitive control centres.^12^ Overall, our result is broadly comparable to a previous case study that showed very little difference between the participants with CBS and other late blind participants without CBS.^22^ In that study, any differences appeared predominantly in white matter and outside of the occipital lobe.

The fixed effects analysis raises interesting questions about the relationship between visual cortex correlations and the strength of hallucinations. For both the LOC and EVC correlations, there was an increased local correlation in those with stronger hallucinations. In contrast, those with no, or weak, correlations showed greater connectivity with regions outside of LOC and EVC. It is not possible to draw strong conclusions since this finding cannot be extrapolated to the general population due to the fixed effects analysis. Nonetheless, this is worthy of further investigation in a larger study.

### Advantages and limitations

The advantages of our study are that participants with CBS are well matched with controls, including their gender, sex, vision and cognitive performance. We can therefore rule out that any neural differences are due to these variables. Age and visual acuity would have affected GABA+ concentrations and visual responsiveness respectively. To our knowledge, we are the first to report in vivo GABA+ concentrations in the visual cortex of people with CBS. The measurements in both early and ventral visual cortex allowed us to directly evaluate cortical excitability at different stages of the visual pathway. Although we did not find any strong evidence supporting the theory, we do not rule out that subtle or transient changes in excitability are related to CBS hallucinations.

Limitations of our study include that the participants had different diagnoses leading to vision loss, which introduced heterogeneity into the cohort. However, this heterogeneity is common across neuroscientific studies of CBS,^25,26,47^ and it is generally accepted that CBS can occur due to different diseases that affect the visual pathway. No sample size or power calculation was performed for this study because no previous study exists that uses MRS to study neurochemicals in participants with CBS. Nevertheless, our sample size of six per group is well within the range of previous fMRI studies involving multiple participants with CBS. Outside of the single case studies,^50^ we are aware of two fMRI studies of groups of patients with CBS. One features eight participants with CBS^26^ and the other five,^47^ although one study employing structural MRI had a higher sample size.^25^ The low sample sizes, and overall sparsity of available studies using fMRI as a methodology, attest to the challenges of using MRI, a technique where age-related contraindications can narrow down the number of eligible participants.

In summary, we evaluated the relationship between neural excitation and CBS by measuring cortical neurochemistry in the visual cortex. Surprisingly, our results show that cortical responses in participants with and without CBS hallucinations are broadly similar. While our study did not provide any consistent evidence in support of the cortical excitability hypothesis, it does not rule out the existence of transient or subtle imbalances in neurochemistry in CBS. We suggest that the cortical mechanisms pre-disposing individuals with vision loss to experience CBS hallucinations are likely to be transient and acting on an otherwise normal visual cortex. In support, a recent Transcranial magnetic stimulation study reported decreases in hallucination frequency with inhibitory stimulation to the visual cortex, which occurred in the absence of any changes in the electrophysiological measure of cortical excitability.^40^ Future investigations could further probe the elusive link between visual hallucinations in CBS and neurochemistry.

## Supplemental Material

sj-docx-1-oed-10.1177_25158414241280201 – Supplemental material for Neurochemistry and functional connectivity in the brain of people with Charles Bonnet syndrome Supplemental material, sj-docx-1-oed-10.1177_25158414241280201 for Neurochemistry and functional connectivity in the brain of people with Charles Bonnet syndrome by Holly Bridge, Abigail Wyllie, Aaron Kay, Bailey Rand, Lucy Starling, Rebecca S. Millington-Truby, William T. Clarke, Jasleen K. Jolly and I. Betina Ip in Therapeutic Advances in Ophthalmology

sj-docx-2-oed-10.1177_25158414241280201 – Supplemental material for Neurochemistry and functional connectivity in the brain of people with Charles Bonnet syndrome Supplemental material, sj-docx-2-oed-10.1177_25158414241280201 for Neurochemistry and functional connectivity in the brain of people with Charles Bonnet syndrome by Holly Bridge, Abigail Wyllie, Aaron Kay, Bailey Rand, Lucy Starling, Rebecca S. Millington-Truby, William T. Clarke, Jasleen K. Jolly and I. Betina Ip in Therapeutic Advances in Ophthalmology

sj-docx-3-oed-10.1177_25158414241280201 – Supplemental material for Neurochemistry and functional connectivity in the brain of people with Charles Bonnet syndrome Supplemental material, sj-docx-3-oed-10.1177_25158414241280201 for Neurochemistry and functional connectivity in the brain of people with Charles Bonnet syndrome by Holly Bridge, Abigail Wyllie, Aaron Kay, Bailey Rand, Lucy Starling, Rebecca S. Millington-Truby, William T. Clarke, Jasleen K. Jolly and I. Betina Ip in Therapeutic Advances in Ophthalmology

## Declarations

**Ethics approval and consent to participate:** All volunteers gave informed and written consent, as approved by the Wales REC 6 Ethics Committee and Health Regulatory Authority (21/WA/0014; Date: 17/02/2021). This study adhered to the Declaration of Helsinki.

**Consent for publication:** Not applicable.

**Availability of data and materials:** MRI data will be made available on zenodo. Analysis scripts will be made available on the last author’s gitlab account.
