# Supplementary material for: Neurochemistry and functional connectivity in the brain of people with Charles Bonnet syndrome
Source: Ther Adv Ophthalmol. 2024 Oct 15;16:25158414241280201. doi: 10.1177/25158414241280201 (PMC11481065; doi:10.1177/25158414241280201)

Minimum Reporting Standards for MRS Checklist

| Site (Name or Number) | 1 |
| --- | --- |
| 1. Hardware |  |
| a. Field strength [T] | 3T |
| b. Manufacturer | Siemens |
| c. Model (software version if available) | Prisma (VE11E) |
| d. RF coils: nuclei (transmit/ receive), number of channels, type, body part | Siemens 64 channel head coil. |
| e. Additional hardware | N/A |
| 2. Acquisition |  |
| a. Pulse sequence | MEGA-PRESS  We used a locally developed MEGA-PRESS sequence, derived from the CMRR spectroscopy package MEGA-PRESS sequence (<https://www.cmrr.umn.edu/spectro/>).  1. Tremblay S, Beaulé V, Proulx S, Lafleur LP, Doyon J, Marjańska M, Théoret H. The use of magnetic resonance spectroscopy as a tool for the measurement of bi-hemispheric transcranial electric stimulation effects on primary motor cortex metabolism. J Vis Exp 2014;93,e51631.  2. Marjańska M, Lehéricy S, Valabrègue R, Popa T, Worbe Y, Russo M, Auerbach EJ, Grabli D, Bonnet C, Gallea C, Coudert M, Yahia-Cherif L, Vidailhet M, Meunier C. Brain dynamic neurochemical changes in dystonic patients: a magnetic resonance spectroscopy study. Mov Disord. 2013;28:201-9. |
| b. Volume of Interest (VOI) locations | Early visual cortex voxel (EVC)  Lateral occipital cortex (LOC) |
| c. Nominal VOI size [cm^3^, mm^3^] | 20x25x25 mm^3^ |
| d. Repetition Time (TR), Echo Time (TE) [ms, s] | TR = 1500 ms  TE = 68 ms |
| e. Total number of Excitations or acquisitions per spectrum  In time series for kinetic studies   1. Number of Averaged spectra (NA) per time-point 2. Averaging method (e.g. block-wise or moving average) 3. Total number of spectra (acquired / in time-series) | 320 spectra in total/ROI composed of 160 edit-off and 160 edit-on spectra |
| f. Additional sequence parameters  (spectral width in Hz, number of spectral points, frequency offsets)  If STEAM:, Mixing Time (TM)  If MRSI: 2D or 3D, FOV in all directions, matrix size, acceleration factors, sampling method | 4000 Hz  2048 points |
| g. Water Suppression Method | VAPOR with additional water suppression using dual-band editing pulse. |
| h. Shimming Method, reference peak, and thresholds for “acceptance of shim” chosen | Automated, vendor supplied 3D GRE *B*_0_ field mapping technique (“GRE Brain”) was used to ensure that vendor-reported full-width-at-half-maximum (FWHM) were below 20 Hz, and that water-unsuppressed MRS-measured FWHM were <12 Hz. |
| i. Triggering or motion correction method  (respiratory, peripheral, cardiac triggering, incl. device used and delays) | None |
| 3. Data analysis methods and outputs |  |
| a. Analysis software | FSL-MRS 2.1.17 |
| b. Processing steps deviating from quoted reference or product | Processing in fsl_mrs_preproc_edit, then fitting using fsl_mrs.  fsl_mrs_preproc_edit \  --data data.nii.gz \  --reference data_wref_phasecorr.nii.gz \  --output /preproc_data \  --leftshift 3 \  --hlsvd \  --report \  --overwrite \  --align_window_dynamic 16 \  --align_ppm_edit 2.5 3.5 \ |
| c. Output measure  (e.g. absolute concentration, institutional units, ratio) Processing steps deviating from quoted reference or product | Tissue corrected absolute concentrations (mMol/kg) |
| d. Quantification references and assumptions, fitting model assumptions | fsl_mrs \  --data diff.nii.gz \  --h2o wref.nii.gz \  --tissue_frac tissue_fraction.json \  --basis uzay_svs_mpress_68_with_db/diff \  --metab_groups sysMM \  --keep GABA GSH Glu Gln NAA NAAG sysMM \  --combine Glu Gln GSH\  --combine GABA sysMM \  --combine NAA NAAG \  --internal_ref NAA \  --baseline_order -1 \  --output /fit_output/data \  --overwrite \  --report \ |
| 4. Data Quality |  |
| a. Reported variables  (SNR, Linewidth (with reference peaks)) | FSL-MRS reported linewidth of the inverted NAA peak (FWHM). |
| b. Data exclusion criteria | None |
| c. Quality measures of postprocessing Model fitting (e.g. CRLB, goodness of fit, SD of residual) | None |
| d. Sample Spectrum | Single subject MRS spectra from EVC and LOC locations are inserted below. |

**CBS Controls**


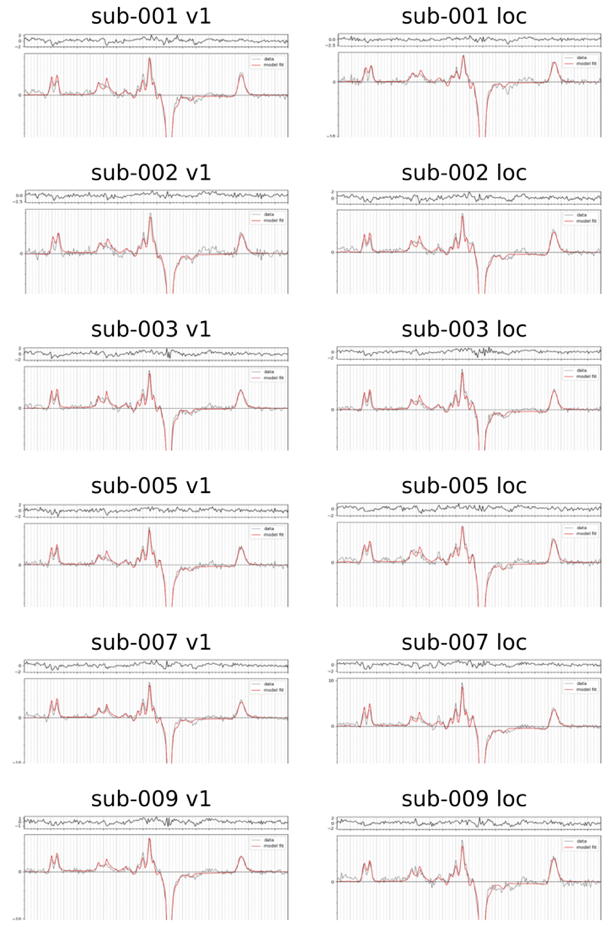

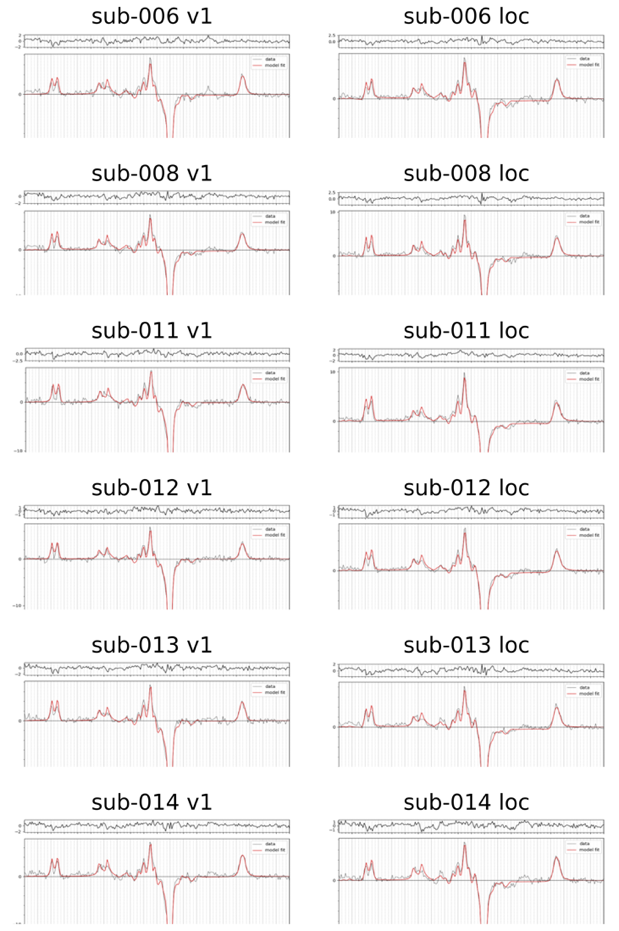

Supplement: sj-docx-1-oed-10.1177_25158414241280201 – Supplemental material for Neurochemistry and functional connectivity in the brain of people with Charles Bonnet syndrome [file sj-docx-1-oed-10.1177_25158414241280201.docx]
