# Supplementary material for: Neurochemistry and functional connectivity in the brain of people with Charles Bonnet syndrome
Source: Ther Adv Ophthalmol. 2024 Oct 15;16:25158414241280201. doi: 10.1177/25158414241280201 (PMC11481065; doi:10.1177/25158414241280201)
Supplement: sj-docx-2-oed-10.1177_25158414241280201 – Supplemental material for Neurochemistry and functional connectivity in the brain of people with Charles Bonnet syndrome [file sj-docx-2-oed-10.1177_25158414241280201.docx]

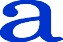


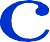

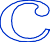

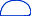

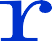

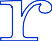


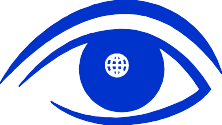


Impact of Vision Impairment Profile (IVI)

**Copyright Centre for Eye Research Australia (CERA)**

**INSTRUCTIONS**

**Please read each question carefully and circle the answer that BEST applies to you. Put one circle on each row.**

**If you use GLASSES, CONTACT LENSES OR MAGNIFIERS for some activities please answer according to how you can see when using them. Here are two examples:**

**In the past month how often has your eyesight made you concerned or worried about…**

|  | **A lot** | **A fair**  **amount** | **A little** | **Not at all** | **Don't do this for**  **other reasons** |
| --- | --- | --- | --- | --- | --- |
| **Crossing the street?** | **0** | **1** | **2** | **3** | **8** |
| **Preparing a meal for yourself?** | **0** | **1** | **2** | **3** | **8** |


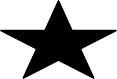
 **PLEASE START HERE AND REMEMBER:**

**Put one circle on each row. Please do not leave any rows blank.**

**Please answer about YOUR eyesight with GLASSES, CONTACT LENSES, or MAGNIFIERS, if you use them. In the PAST MONTH, how much has YOUR EYESIGHT INTERFERED with the following activities:**

|  | **A lot** | **A fair**  **amount** | **A little** | **Not at**  **all** | **Don't do this for**  **other reasons** | **Domain** |
| --- | --- | --- | --- | --- | --- | --- |
| **1. Your ability to see and enjoy T.V?** | **0** | **1** | **2** | **3** | **8** | **Reading and accessing**  **information** |
| **2. Taking part in recreational activities such as bowling, walking or golf?** | **0** | **1** | **2** | **3** | **8** | **Mobility and independence** |
| **3. Shopping? (finding what you want and paying for it)** | **0** | **1** | **2** | **3** | **8** | **Reading and accessing**  **information** |
| **4. Visiting friends or family?** | **0** | **1** | **2** | **3** | **8** | **Mobility and**  **independence** |
| **5. Recognising or meeting people?** | **0** | **1** | **2** | **3** | **8** | **Reading and accessing**  **information** |
| **6. Generally looking after your appearance? (face, hair, clothing etc.)** | **0** | **1** | **2** | **3** | **8** | **Reading and**  **accessing information** |

| **7. Opening packaging? (for example, around food, medicines)** | **0** | **1** | **2** | **3** | **8** | **Reading and accessing**  **information** |
| --- | --- | --- | --- | --- | --- | --- |
| **8. Reading labels or instructions on medicines?** | **0** | **1** | **2** | **3** | **8** | **Reading and accessing**  **information** |
| **9. Operating household appliances and the telephone?** | **0** | **1** | **2** | **3** | **8** | **Reading and**  **accessing information** |
| **10. How much has your eyesight interfered with getting about outdoors? (on the pavement or crossing the street)** | **0** | **1** | **2** | **3** | **8** | **Mobility and independence** |
| **11. In the past month, how often has your eyesight made you go carefully to avoid**  **falling or tripping?** | **0** | **1** | **2** | **3** | **8** | **Mobility and independence** |
| **12. In general, how much has your**  **eyesight interfered with travelling or using transport? (bus & train)** | **0** | **1** | **2** | **3** | **8** | **Mobility and independence** |
| **13. Going down steps, stairs, or curbs?** | **0** | **1** | **2** | **3** | **8** | **Mobility and**  **independence** |

**In the PAST MONTH, how much has YOUR EYESIGHT INTERFERED with the following activities**

|  | **A lot** | **A fair amount** | **Not at all** | **Don't do this for**  **other reasons** |  |
| --- | --- | --- | --- | --- | --- |
| **14. Reading ordinary size print? (for example newspapers)** | **0** | **1** | **2** | **8** | **Reading and accessing information** |
| **15. Getting information that you need?** | **0** | **1** | **2** | **8** | **Reading and accessing information** |

**Please answer about YOUR eyesight with GLASSES, CONTACT LENSES or MAGNIFIERS, if you use them.**

**In the PAST MONTH, how often has YOUR EYESIGHT MADE YOU CONCERNED OR WORRIED about the following:**

|  | **A lot of the**  **time** | **A fair amount**  **of the time** | **A little of the time** | **Not at all** | **Domain** |
| --- | --- | --- | --- | --- | --- |
| **16. Your general safety at home?** | **0** | **1** | **2** | **3** | **Mobility and**  **independence** |
| **17. Spilling or breaking things?** | **0** | **1** | **2** | **3** | **Mobility and**  **independence** |
| **18. Your general safety when out of your home?** | **0** | **1** | **2** | **3** | **Mobility and independence** |
| **19. In the past month, how often has your eyesight stopped you doing the things you want**  **to do?** | **0** | **1** | **2** | **3** | **Mobility and independence** |
| **20. In the past month, how often have you needed help from other people because of your**  **eyesight?** | **0** | **1** | **2** | **3** | **Mobility and independence** |

**Please answer about YOUR eyesight with GLASSES, CONTACT LENSES or MAGNIFIERS, if you use them.**

**Think about how YOUR eyesight has made you FEEL in the PAST MONTH.**

|  | **A lot of the**  **time** | **A fair amount of**  **the time** | **A little of the time** | **Not at all** | **Domain** |
| --- | --- | --- | --- | --- | --- |
| **21. Have you felt embarrassed because of your**  **eyesight?** | **0** | **1** | **2** | **3** | **Emotional**  **well-being** |
| **22. Have you felt frustrated or annoyed**  **because of your eyesight?** | **0** | **1** | **2** | **3** | **Emotional**  **well-being** |
| **23. Have you felt lonely or isolated because of**  **your eyesight?** | **0** | **1** | **2** | **3** | **Emotional**  **well-being** |
| **24. Have you felt sad or low because of your**  **eyesight?** | **0** | **1** | **2** | **3** | **Emotional**  **well-being** |
| **25. In the past month, how often have you worried about your eyesight getting worse?** | **0** | **1** | **2** | **3** | **Emotional well-being** |
| **26. In the past month how often has your eyesight made you concerned or worried about**  **coping with everyday life?** | **0** | **1** | **2** | **3** | **Emotional well-being** |
| **27. Have you felt like a nuisance or a burden**  **because of your eyesight?** | **0** | **1** | **2** | **3** | **Emotional**  **well-being** |
| **28. In the past month, how much has your**  **eyesight interfered with your life in general?** | **0** | **1** | **2** | **3** | **Emotional**  **well-being** |

**Please check that you have answered all the questions and Thank you!**

**The University of Miami Parkinson’s disease Hallucinations Questionnaire (UM-PDHQ)**

Patient identifier: DATE:

|  | **Question** | **A:Features/Comments** | **B:Score (circle appropriate)** |
| --- | --- | --- | --- |
| **Severity of hallucinations** | 1. Do you experience hallucinations? (Have you noticed anything unusual about your vision? Have you had any unusual visual experiences? Or ever see/hear/feel/smell/taste things that are not really there or that other  people do not see?) | Type: (mark appropriate)   1. Visual 2. Auditory 3. Somatic/Cutaneous 4. Gustatory 5. Olfactory   (assess each separately) | 1. No hallucinations (skip to Annex) 2. One type only 3. Combination   **C:** Not within the past month, but it has happened in the past |
|  | 2. How often do you experience hallucinations? |  | 0 = Only a few times  1 = Occasionally (less than once a week, but continuously)  2 = Often (about once per week)  3 = Frequently (several times per week but < than once per day)  4 = Very frequently (≥once per day) |
|  | 3. On average, how long do the experiences last? |  | 0 = Short Duration (< 1sec)  1 = Medium Duration (< 10secs)  2 = Prolonged Duration (> 10secs) |
|  | 4. Do you think what you are seeing/experiencing is real? |  | 0 = Not real  1 = Sometimes real  2 = Always real |
|  | 5. How many types of images/sensations do you  experience? |  | 1 = One  2 = Few (2 or 3)  3 = Several (more than 3) |
|  | 6. How severe/emotionally distressing do you find these images/sensations or visions? |  | 0 = No effect/Friendly  1 = Mildly – produce little distress  2 = Moderately – produce distress and are disturbing and disruptive  3 = Severely – very disturbing  (medications may be required) |
|  | Total Score (min = 0; max = 14) |  |  |

# Comments:

| **Quality of hallucinations** | **Please circle the appropriate answer and provide information** | |
| --- | --- | --- |
|  | 7. Have you been diagnosed with any eye disease? (i.e. near or far sight problems, double vision, cataract,  glaucoma, retinitis, retinal detachment, diabetic or hypertensive eye disease) | Yes (please describe) No |
|  | 8. What are your current medications? | *Complete medication data on page 4.* |
|  | 9. Was there a recent change in your  treatment? Please describe. | Yes (please describe)  No |
|  | 10. Was this change related to the appearance or change in the characteristics of hallucinations? | Yes No  I cannot tell  N/A |
|  | 11. Do you experience hallucinations while “on” or “off”? | On Off  Anytime-not related to on-offs |
|  | 12. What do you normally see/feel/hear/smell/taste?  If not visual describe here:  Voices, Music, tastes, smells, skin related: | Not formed/cannot describe Whole Faces  Fragmented faces □ Familiar  Whole people □ Unfamiliar  Animals Insects/reptiles  Objects |
|  | 13. Is there anything you can do to make  the images/sensations disappear? | Yes  No |
|  | 14. At what time of the day or under which lighting conditions do you experience hallucinations | 1. Specific time During the day/Bright During the night/Dark Dim 2. Anytime |
|  | 15. Do the images ever make any sound or noise (for visual hallucinations)? | Yes No  N/A (for non-visual hallucinations) |
|  | 16. Do images move (for visual hallucinations)? | Yes No  N/A (for non-visual hallucinations) |
|  | 17. Are the images normal size? | Yes  No, smaller than normal No, larger than normal  N/A (for non-visual hallucinations) |
|  | 18. Are the images transparent or solid? | Transparent Solid  N/A (for non-visual hallucinations) |
|  | 19. Are the images colored? | Yes  No, (black and white)  N/A (for non-visual hallucinations) |
|  | 20. Is the onset of hallucinations gradual or sudden? | Gradual (appear-disappear slowly)  Sudden (appear-disappear suddenly) I cannot tell |

**MONTREAL COGNITIVE ASSESSMENT (MoCA®)**

Version 8.3 BLIND English

**NAME: EDUCATION :**

**Sex : Date of birth :**

**DATE :**

| **MEMORY** |  | LEG | COTTON | SCHOOL | TOMATO | WHITE | **POINTS** |
| --- | --- | --- | --- | --- | --- | --- | --- |
| Read list of words, subject must repeat them. Do 2 trials, even if 1st trial is successful.  Do a recall after 5 minutes. | 1st TRIAL |  |  |  |  |  | **NO**  **POINTS** |
|  | 2nd TRIAL |  |  |  |  |  |  |
| **ATTENTION** | Subject has to repeat in the forward order. [ ] 2 4 8 1 5 | | | | | | /2 |
| Read list of digits (1 digit / sec.). Subject has to repeat in the backward order. [ ] 4 2 7 | | | | | | |  |
| Read list of letters. The subject must tap at each letter A. No points if ≥ 2 errors  **[ ] F B A C M N A A J K L B A F A K D E A A A J A M O F A A B** | | | | | | | /1 |
| Seria 7 subtraction starting at 60 [ ] 53 [ ] 46 [ ] 39 [ ] 32 [ ] 25  4 or 5 correct subtractions: **3 pts,** 2 or 3 correct: **2 pts,** 1 correct: **1 pt,** 0 correct: **0 pt** | | | | | | | /3 |
| **LANGUAGE** | **The child walked his dog in the park after midnight. [ ]** | | | | | | /2 |
| **Repeat: The artist finished his painting at the right moment for the exhibition. [ ]** | | | | | | |  |
| **Fluency: Name maximum number of words in one minute that begin with the letter B.**  [ ] ( N ≥ 11words) | | | | | | | /1 |
| **ABSTRACTION** | **[ ] hammer - screwdriver** | | | | | | /2 |
| Similarity between e.g. orange - banana = fruit **[ ] matches - lamp** | | | | | | |  |
| **DELAYED RECALL** | Has to recall words WITH NO CUE | LEG  [ ] | COTTON  [ ] | SCHOOL  [ ] | TOMATO WHITE | | /5 |
| Memory ( MIS) X3  Index X2  Score X1 |  |  |  |  | [ ] | [ ] |  |
|  | Category cue |  |  |  |  |  | **NO**  **POINTS** |
|  | Multiple  choice cue |  |  |  |  |  |  |
| **ORIENTATION** | [ ] Date [ ] Month [ ] Year [ ] Day [ ] Place [ ] City | | | | | | /6 |
| **© Z. Nasreddine MD** [**www.mocatest.org**](http://www.mocatest.org/)  Administered by : Add 1 point if ≤ 12 yr edu | | | | | TOTAL | | /22 |

**MIS: /15 (Normal ≥ 19/22)**

**Training and Certification are required to ensure accuracy**

DIRECTIONS FOR ADMINISTRATION:

- Do not use the term ‘hallucination’, unless term is first used by the participant. Instead, use the term ‘experience’.
- There is a series of screening questions which refer to the presence/absence of visual hallucinations and other experiences. Whenever the answer ‘Yes’ is given, record in as much detail as possible in the box below what they have seen. Prompt participant to report if what they saw moved, its colour, its size, its contour, its shape and where in the field of view it was seen. After description, ask the specific sub-questions for further details about the experience.
- If no screening question is endorsed, miss the following section asking the participant to choose their most distressing visual experience, and ask the final screening question about auditory hallucinations.
- The opening question is for the participant to get comfortable talking about their experience (so write description in relevant screening section). Even if they say no, go through remaining screening items.

**PRESENCE**

*Presence of somebody/something next to you*

**GO TO Q.3**

**ILLUSION**

*Faces/objects in patterns/surfaces/textures*

**GO TO Q.3**

1. **DO YOU FEEL LIKE YOUR EYES EVER PLAY TRICKS ON YOU? HAVE YOU EVER SEEN SOMETHING (OR THINGS) THAT OTHER PEOPLE COULD NOT SEE?**

No (0)

Yes (1)

1. **IF YES, PLEASE DESCRIBE WHAT YOU HAVE SEEN:**

**GO TO Q.4**

**GO TO Q.5**

**SIMPLE**

*dots/flashes of light*

**GO TO Q.6- Q.10**

**COMPLEX**

*patterns/faces/objects/people/animals*

**PASSAGE**

*Animals/People moving past & disappeared when looked at*

**GO TO Q.11- Q.12**

| M | M | / | Y | Y | Y | Y |
| --- | --- | --- | --- | --- | --- | --- |

| M | M | / | Y | Y | Y | Y |
| --- | --- | --- | --- | --- | --- | --- |

|  |
| --- |
|  |
|  |
|  |

| **3. HAVE YOU SEEN FACES OR OBJECTS IN PATTERNS, SURFACES, OR TEXTURES?**  No (0) **GO TO Q.4**  Yes (1) | | |
| --- | --- | --- |
| **PLEASE DESCRIBE WHAT YOU HAVE SEEN:** does it move, colour, size, contour, shape, field of view | | |
| **A. WHEN DID THIS FIRST START?**  *If date unknown: 01/1900* | | |
| **B. WHEN DID THIS LAST HAPPEN?**  *If date unknown: 01/1900* | | |
| **C. APPROXIMATELY HOW LONG DO THESE EXPERIENCES USUALLY LAST?**  Seconds (1) ***specify***  Minutes (2) ***specify***  Hours (3) ***specify***  Continuous while awake (4) | | |
| **D. HOW OFTEN DO THEY USUALLY OCCUR?** | | |
| Less than every few months  Every few months Every few weeks Every few days Every few hours Every few minutes Every few seconds  Continuously- present throughout the day |  | (1)  (2)  (3)  (4)  (5)  (6)  (7)  (8) |
|  |  |  |
|  |  |  |
|  |  |  |
|  |  |  |
|  |  |  |
|  |  |  |
|  |  |  |
|  | | |

|  |  |  |  |  |
| --- | --- | --- | --- | --- |

|  |  |  |  |  |
| --- | --- | --- | --- | --- |

| **E. IN A TYPICAL MONTH, HOW MANY EXPERIENCES WOULD YOU HAVE?** |
| --- |
| **F. AND IN THIS TYPICAL MONTH, HOW MANY DAYS WOULD YOU HAVE THESE EXPERIENCES?** |
| **G. IN THE LAST 3 MONTHS HOW MANY EXPERIENCES HAVE YOU HAD?** |
| **H. AND IN THE LAST 3 MONTHS, HOW MANY DAYS WOULD YOU HAVE THESE EXPERIENCES?** |
| **I. IS THIS EXPERIENCE ASSOCIATED WITH FALLING ASLEEP OR WAKING UP?**  Never (0) Sometimes (1) Always (2) |
| **J. AT WHAT TIME OF THE DAY DOES THIS EXPERIENCE USUALLY OCCUR?**  Night (0) Day time (1) Any time (2) |
| **K. DOES THE EXPERIENCE EVER SPEAK OR MAKE NOISES?**  Never (0) Sometimes (1) Always (2) |
| **L. IS THIS EXPERIENCE ASSOCIATED WITH AN ODD SMELL OR TASTE?**  Never (0) Sometimes (1) Always (2) |
| **M. DOES IT EVER FEEL LIKE IT IS TOUCHING YOU?**  Never (0) Sometimes (1) Always (2) |
| **N. WHILST YOU ARE HAVING THE EXPERIENCE DO YOU EVER BELIEVE IT IS REAL?**  Never (0) Sometimes (1) Always (2) |
| **O. DO YOU EVER ACT ON THE EXPERIENCE?**  Never (0) Sometimes (1) Always (2) |
| **P. DO YOU HAVE AN EXPLANATION OF THESE EXPERIENCES THAT OTHERS SAY ARE NOT TRUE OR REAL?**  Never (0) Sometimes (1) Always (2) |

| M | M | / | Y | Y | Y | Y |
| --- | --- | --- | --- | --- | --- | --- |

| M | M | / | Y | Y | Y | Y |
| --- | --- | --- | --- | --- | --- | --- |

|  |
| --- |
|  |
|  |
|  |

| **4. HAVE YOU EVER HAD THE FEELING OF THE PRESENCE OF SOMEBODY, OR SOMETHING, NEXT TO YOU?**  No (0) **GO TO Q.5**  Yes (1) | | |
| --- | --- | --- |
| **PLEASE DESCRIBE WHAT YOU HAVE SEEN:** does it move, colour, size, contour, shape, field of view | | |
| **A. WHEN DID THIS FIRST START?**  *If date unknown: 01/1900* | | |
| **B. WHEN DID THIS LAST HAPPEN?**  *If date unknown: 01/1900* | | |
| **C. APPROXIMATELY HOW LONG DO THESE EXPERIENCES USUALLY LAST?**  Seconds (1) ***specify***  Minutes (2) ***specify***  Hours (3) ***specify***  Continuous while awake (4) | | |
| **D. HOW OFTEN DO THEY USUALLY OCCUR?** | | |
| Less than every few months  Every few months Every few weeks Every few days Every few hours Every few minutes Every few seconds  Continuously- present throughout the day |  | (1)  (2)  (3)  (4)  (5)  (6)  (7)  (8) |
|  |  |  |
|  |  |  |
|  |  |  |
|  |  |  |
|  |  |  |
|  |  |  |
|  |  |  |

|  |  |  |  |  |
| --- | --- | --- | --- | --- |

|  |  |  |  |  |
| --- | --- | --- | --- | --- |

| **E. IN A TYPICAL MONTH, HOW MANY EXPERIENCES WOULD YOU HAVE?** |
| --- |
| **F. AND IN THIS TYPICAL MONTH, HOW MANY DAYS WOULD YOU HAVE THESE EXPERIENCES?** |
| **G. IN THE LAST 3 MONTHS HOW MANY EXPERIENCES HAVE YOU HAD?** |
| **H. AND IN THE LAST 3 MONTHS, HOW MANY DAYS WOULD YOU HAVE THESE EXPERIENCES?** |
| **I. IS THIS EXPERIENCE ASSOCIATED WITH FALLING ASLEEP OR WAKING UP?**  Never (0) Sometimes (1) Always (2) |
| **J. AT WHAT TIME OF THE DAY DOES THIS EXPERIENCE USUALLY OCCUR?**  Night (0) Day time (1) Any time (2) |
| **K. DOES THE EXPERIENCE EVER SPEAK OR MAKE NOISES?**  Never (0) Sometimes (1) Always (2) |
| **L. IS THIS EXPERIENCE ASSOCIATED WITH AN ODD SMELL OR TASTE?**  Never (0) Sometimes (1) Always (2) |
| **M. DOES IT EVER FEEL LIKE IT IS TOUCHING YOU?**  Never (0) Sometimes (1) Always (2) |
| **N. WHILST YOU ARE HAVING THE EXPERIENCE DO YOU EVER BELIEVE IT IS REAL?**  Never (0) Sometimes (1) Always (2) |
| **O. DO YOU EVER ACT ON THE EXPERIENCE?**  Never (0) Sometimes (1) Always (2) |
| **P. DO YOU HAVE AN EXPLANATION OF THESE EXPERIENCES THAT OTHERS SAY ARE NOT TRUE OR REAL?**  Never (0) Sometimes (1) Always (2) |

| M | M | / | Y | Y | Y | Y |
| --- | --- | --- | --- | --- | --- | --- |

| M | M | / | Y | Y | Y | Y |
| --- | --- | --- | --- | --- | --- | --- |

|  |
| --- |
|  |
|  |
|  |

| **5. HAVE YOU SEEN DOTS, FLASHES OF LIGHT, OR SIMILAR THAT WERE NOT THERE?**  No (0) **GO TO Q.6**  Yes (1) | | |
| --- | --- | --- |
| **PLEASE DESCRIBE WHAT YOU HAVE SEEN:** does it move, colour, size, contour, shape, field of view | | |
| **A. WHEN DID THIS FIRST START?**  *If date unknown: 01/1900* | | |
| **B. WHEN DID THIS LAST HAPPEN?**  *If date unknown: 01/1900* | | |
| **C. APPROXIMATELY HOW LONG DO THESE EXPERIENCES USUALLY LAST?**  Seconds (1) ***specify***  Minutes (2) ***specify***  Hours (3) ***specify***  Continuous while awake (4) | | |
| **D. HOW OFTEN DO THEY USUALLY OCCUR?** | | |
| Less than every few months  Every few months Every few weeks Every few days Every few hours Every few minutes Every few seconds  Continuously- present throughout the day |  | (1)  (2)  (3)  (4)  (5)  (6)  (7)  (8) |
|  |  |  |
|  |  |  |
|  |  |  |
|  |  |  |
|  |  |  |
|  |  |  |
|  |  |  |

|  |  |  |  |  |
| --- | --- | --- | --- | --- |

|  |  |  |  |  |
| --- | --- | --- | --- | --- |

| **E. IN A TYPICAL MONTH, HOW MANY EXPERIENCES WOULD YOU HAVE?** |
| --- |
| **F. AND IN THIS TYPICAL MONTH, HOW MANY DAYS WOULD YOU HAVE THESE EXPERIENCES?** |
| **G. IN THE LAST 3 MONTHS HOW MANY EXPERIENCES HAVE YOU HAD?** |
| **H. AND IN THE LAST 3 MONTHS, HOW MANY DAYS WOULD YOU HAVE THESE EXPERIENCES?** |
| **I. IS THIS EXPERIENCE ASSOCIATED WITH FALLING ASLEEP OR WAKING UP?**  Never (0) Sometimes (1) Always (2) |
| **J. AT WHAT TIME OF THE DAY DOES THIS EXPERIENCE USUALLY OCCUR?**  Night (0) Day time (1) Any time (2) |
| **K. DOES THE EXPERIENCE EVER SPEAK OR MAKE NOISES?**  Never (0) Sometimes (1) Always (2) |
| **L. IS THIS EXPERIENCE ASSOCIATED WITH AN ODD SMELL OR TASTE?**  Never (0) Sometimes (1) Always (2) |
| **M. DOES IT EVER FEEL LIKE IT IS TOUCHING YOU?**  Never (0) Sometimes (1) Always (2) |
| **N. WHILST YOU ARE HAVING THE EXPERIENCE DO YOU EVER BELIEVE IT IS REAL?**  Never (0) Sometimes (1) Always (2) |
| **O. DO YOU EVER ACT ON THE EXPERIENCE?**  Never (0) Sometimes (1) Always (2) |
| **P. DO YOU HAVE AN EXPLANATION OF THESE EXPERIENCES THAT OTHERS SAY ARE NOT TRUE OR REAL?**  Never (0) Sometimes (1) Always (2) |

| If participant says ‘Yes’ to any of the following 5 experiences (complex hallucinations- patterns, faces,  objects, people or animals), ask them to complete the relevant sub-questions on the most prevalent experience. |
| --- |

**6. HAVE YOU SEEN PATTERNS, LATTICES, BRICKWORK, CHEQUER-BOARDS OR SIMILAR THAT WERE NOT THERE?**

No (0)

Yes (1)

**PLEASE DESCRIBE WHAT YOU HAVE SEEN:** does it move, colour, size, contour, shape, field of view

**GO TO Q.7**

| **7. HAVE YOU EVER SEEN FACES WITHOUT A BODY?**  No (0) **GO TO Q.8**  Yes (1) |
| --- |
| **PLEASE DESCRIBE WHAT YOU HAVE SEEN:** does it move, colour, size, contour, shape, field of view |
| **8. HAVE YOU SEEN OBJECTS THAT WERE NOT THERE?** |

| No (0) **GO TO Q.9**  Yes (1) |
| --- |
| **PLEASE DESCRIBE WHAT YOU HAVE SEEN:** does it move, colour, size, contour, shape, field of view |

**9. HAVE YOU EVER SEEN PEOPLE THAT WERE NOT THERE?** (make sure that this is not a passage

hallucination (see people moving past and when looked at disappeared, Q. 11))

No

Yes

(0)

(1)

**PLEASE DESCRIBE WHAT YOU HAVE SEEN:** does it move, colour, size, contour, shape, field of view

**GO TO Q.10**

**A. OUT OF THE LAST EXPERIENCES YOU JUST DESCRIBED** (specify: PATTERNS, FACES, OBJECTS, PEOPLE, ANIMALS) **WHICH DID YOU HAVE MOST OFTEN?**

Patterns (0)

Faces (1)

Objects (2)

People (3)

Animals (4)

**B. WHEN DID THIS FIRST START?**

*If date unknown: 01/1900*

**C. WHEN DID THIS LAST HAPPEN?**

**10. HAVE YOU EVER SEEN ANIMALS THAT WERE NOT THERE** (make sure that this is not a passage

hallucination (see animals moving past and when looked at disappeared, Q. 12))

No

Yes

(0)

(1)

**PLEASE DESCRIBE WHAT YOU HAVE SEEN:** does it move, colour, size, contour, shape, field of view

**GO TO Q.11**

| If participant has said ‘Yes’ to any of the past 5 experiences (complex hallucinations- patterns, faces, objects, people or animals), ask them to complete the next sub-questions on the most prevalent  experience. |
| --- |

|  |
| --- |
|  |
|  |
|  |
|  |

| M | M | / | Y | Y | Y | Y |
| --- | --- | --- | --- | --- | --- | --- |

| M | M | / | Y | Y | Y | Y |
| --- | --- | --- | --- | --- | --- | --- |

|  |  |  |  |  |
| --- | --- | --- | --- | --- |

|  |  |  |  |  |
| --- | --- | --- | --- | --- |

| *If date unknown: 01/1900* | | |
| --- | --- | --- |
| **D. APPROXIMATELY HOW LONG DO THESE EXPERIENCES USUALLY LAST?** | | |
| Seconds Minutes Hours  Continuous while awake |  | 1. ***specify*** 2. ***specify*** 3. ***specify*** 4. ​ |
|  |  |  |
|  |  |  |
|  |  |  |
| **E. HOW OFTEN DO THEY USUALLY OCCUR?** | | |
| Less than every few months  Every few months Every few weeks Every few days Every few hours Every few minutes Every few seconds  Continuously- present throughout the day |  | (1)  (2)  (3)  (4)  (5)  (6)  (7)  (8) |
|  |  |  |
|  |  |  |
|  |  |  |
|  |  |  |
|  |  |  |
|  |  |  |
|  |  |  |
| **F. IN A TYPICAL MONTH, HOW MANY EXPERIENCES WOULD YOU HAVE?** | | |
| **G. AND IN THIS TYPICAL MONTH, HOW MANY DAYS WOULD YOU HAVE THESE EXPERIENCES?** | | |
| **H. IN THE LAST 3 MONTHS, HOW MANY EXPERIENCES HAVE YOU HAD?** | | |
| **I. AND IN THE LAST 3 MONTHS, HOW MANY DAYS WOULD YOU HAVE THESE EXPERIENCES?** | | |
| **J. IS THIS EXPERIENCE ASSOCIATED WITH FALLING ASLEEP OR WAKING UP?**  Never (0) Sometimes (1) Always (2) | | |
| **K. AT WHAT TIME OF THE DAY DOES THIS EXPERIENCE USUALLY OCCUR?**  Night (0) Day time (1) Any time (2) | | |
| **L. DOES THE EXPERIENCE EVER SPEAK OR MAKE NOISES?**  Never (0) Sometimes (1) Always (2) | | |

|  | |
| --- | --- |
| **M. IS THIS EXPERIENCE ASSOCIATED WITH AN ODD SMELL OR TASTE?**  Never (0) Sometimes (1) Always | (2) |
| **N. DOES IT EVER FEEL LIKE IT IS TOUCHING YOU?**  Never (0) Sometimes (1) Always | (2) |
| **O. WHILST YOU ARE HAVING THE EXPERIENCE DO YOU EVER BELIEVE IT IS REAL?**  Never (0) Sometimes (1) Always | (2) |
| **P. DO YOU EVER ACT ON THE EXPERIENCE?**  Never (0) Sometimes (1) Always | (2) |
| **Q. DO YOU HAVE AN EXPLANATION OF THESE EXPERIENCES THAT OTHERS SAY ARE NOT TRUE OR REAL?**  Never (0) Sometimes (1) Always (2) | |

**11. HAVE YOU EVER SEEN PEOPLE MOVING PAST YOU AND THAT DISAPPEARED WHEN YOU LOOKED AT THEM?**

No (0)

Yes (1)

**PLEASE DESCRIBE WHAT YOU HAVE SEEN:** does it move, colour, size, contour, shape, field of view

**GO TO Q.12**

| M | M | / | Y | Y | Y | Y |
| --- | --- | --- | --- | --- | --- | --- |

|  |  |  |  |  |  |  |
| --- | --- | --- | --- | --- | --- | --- |

**12. HAVE YOU EVER SEEN ANIMALS MOVING PAST YOU AND THAT DISAPPEARED WHEN YOU LOOKED AT THEM?**

No (0)

Yes (1)

**PLEASE DESCRIBE WHAT YOU HAVE SEEN:** does it move, colour, size, contour, shape, field of view

**GO TO Q.13**

| If participant has said ‘Yes’ to any of the past 2 experiences (passage hallucinations) ask them to  complete the next sub-questions on the most prevalent experience. |
| --- |

| **A. OUT OF THE LAST EXPERIENCES YOU JUST DESCRIBED** (specify: seeing people or animals moving),  **WHICH DID YOU HAVE MOST OFTEN?**  People (0)  Animals (1) |
| --- |
| **B. WHEN DID THIS FIRST START?**  *If date unknown: 01/1900* |
| **C. WHEN DID THIS LAST HAPPEN?**  *If date unknown: 01/1900* M M / Y Y Y Y |

|  |
| --- |
|  |
|  |
|  |
|  |
|  |
|  |
|  |

|  |  |  |  |  |
| --- | --- | --- | --- | --- |

|  |  |  |  |  |
| --- | --- | --- | --- | --- |

|  | | |
| --- | --- | --- |
| **D. APPROXIMATELY HOW LONG DO THESE EXPERIENCES USUALLY LAST?** | | |
| Seconds Minutes Hours  Continuous while awake |  | 1. ***specify*** 2. ***specify*** 3. ***specify*** 4. ​ |
|  |  |  |
|  |  |  |
|  |  |  |
| **E. HOW OFTEN DO THEY USUALLY OCCUR?**  Less than every few months (1) Every few months (2)  Every few weeks (3)  Every few days (4)  Every few hours (5)  Every few minutes (6)  Every few seconds (7) Continuously- present throughout the day (8) | | |
| **F. IN A TYPICAL MONTH, HOW MANY EXPERIENCES WOULD YOU HAVE?** | | |
| **G. AND IN THIS TYPICAL MONTH, HOW MANY DAYS WOULD YOU HAVE THESE EXPERIENCES?** | | |
| **H. IN THE LAST 3 MONTHS, HOW MANY EXPERIENCES HAVE YOU HAD?** | | |
| **I. AND IN THE LAST 3 MONTHS, HOW MANY DAYS WOULD YOU HAVE THESE EXPERIENCES?** | | |
| **J. IS THIS EXPERIENCE ASSOCIATED WITH FALLING ASLEEP OR WAKING UP?**  Never (0) Sometimes (1) Always (2) | | |
| **K. AT WHAT TIME OF THE DAY DOES THIS EXPERIENCE USUALLY OCCUR?**  Night (0) Day time (1) Any time (2) | | |
| **L. DOES THE EXPERIENCE EVER SPEAK OR MAKE NOISES?**  Never (0) Sometimes (1) Always (2) | | |

|  | |
| --- | --- |
| **M. IS THIS EXPERIENCE ASSOCIATED WITH AN ODD SMELL OR TASTE?**  Never (0) Sometimes (1) Always | (2) |
| **N. DOES IT EVER FEEL LIKE IT IS TOUCHING YOU?**  Never (0) Sometimes (1) Always | (2) |
| **O. WHILST YOU ARE HAVING THE EXPERIENCE DO YOU EVER BELIEVE IT IS REAL?**  Never (0) Sometimes (1) Always | (2) |
| **P. DO YOU EVER ACT ON THE EXPERIENCE?**  Never (0) Sometimes (1) Always | (2) |
| **Q. DO YOU HAVE AN EXPLANATION OF THESE EXPERIENCES THAT OTHERS SAY ARE NOT TRUE OR REAL?**  Never (0) Sometimes (1) Always (2) | |

**13. HAVE YOU EVER HAD ANY OTHER VISUAL EXPERIENCE?**

No

(0)

Yes

(1)

**PLEASE DESCRIBE WHAT YOU HAVE SEEN:** does it move, colour, size, contour, shape, field of view

**GO TO Q.14**

1. **WHEN DID THIS FIRST START?**

*If date unknown: 01/1900*

| M | M | / | Y | Y | Y | Y |
| --- | --- | --- | --- | --- | --- | --- |

1. **WHEN DID THIS LAST HAPPEN?**

*If date unknown: 01/1900*

| M | M | / | Y | Y | Y | Y |
| --- | --- | --- | --- | --- | --- | --- |

1. **APPROXIMATELY HOW LONG DO THESE EXPERIENCES USUALLY LAST?**

Seconds Minutes Hours

1. ***specify***
2. ***specify***
3. ***specify***

Continuous while awake

1. **HOW OFTEN DO THEY USUALLY OCCUR?**

Less than every few months

Every few months Every few weeks Every few days Every few hours Every few minutes Every few seconds

Continuously- present throughout the day

(4)

(1)

(2)

(3)

(4)

(5)

(6)

(7)

(8)

1. **IN A TYPICAL MONTH, HOW MANY EXPERIENCES WOULD YOU HAVE?**

|  |  |  |  |  |
| --- | --- | --- | --- | --- |

1. **AND IN THIS TYPICAL MONTH, HOW MANY DAYS WOULD YOU HAVE THESE EXPERIENCES?**
2. **IN THE LAST 3 MONTHS, HOW MANY EXPERIENCES HAVE YOU HAD?**

|  |  |  |  |  |
| --- | --- | --- | --- | --- |

1. **AND IN THE LAST 3 MONTHS, HOW MANY DAYS WOULD YOU HAVE THESE EXPERIENCES?**
2. **IS THIS EXPERIENCE ASSOCIATED WITH FALLING ASLEEP OR WAKING UP?**

**14. OUT OF ALL THE EXPERIENCES YOU JUST DESCRIBED** (specify: INSERT RELEVANT DESCRIPTION),

**WHICH DID YOU FIND MOST DISTRESSING?**

Illusions (0)

Presence (1)

Simple (2)

Complex (3)

Passage (4)

| Never (0) Sometimes (1) Always (2) |
| --- |
| **J. AT WHAT TIME OF THE DAY DOES THIS EXPERIENCE USUALLY OCCUR?**  Night (0) Day time (1) Any time (2) |
| **K. DOES THE EXPERIENCE EVER SPEAK OR MAKE NOISES?**  Never (0) Sometimes (1) Always (2) |
| **L. IS THIS EXPERIENCE ASSOCIATED WITH AN ODD SMELL OR TASTE?**  Never (0) Sometimes (1) Always (2) |
| **M. DOES IT EVER FEEL LIKE IT IS TOUCHING YOU?**  Never (0) Sometimes (1) Always (2) |
| **N. WHILST YOU ARE HAVING THE EXPERIENCE DO YOU EVER BELIEVE IT IS REAL?**  Never (0) Sometimes (1) Always (2) |
| **O. DO YOU EVER ACT ON THE EXPERIENCE?**  Never (0) Sometimes (1) Always (2) |
| **P. DO YOU HAVE AN EXPLANATION OF THESE EXPERIENCES THAT OTHERS SAY ARE NOT TRUE OR REAL?**  Never (0) Sometimes (1) Always (2) |

| If participant has said ‘Yes’ to any of the past visual experiences ask them to complete the next sub- questions on the most distressing experience.  If participant has not said ‘Yes’ to any of the past experiences, go to Q. 15. |
| --- |

|  |
| --- |
|  |
|  |
|  |
|  |

| Other | (5) |  |  |
| --- | --- | --- | --- |
| **A. FROM 0 TO 10, HOW FRIGHTENING OR DISTRESSING WAS THIS EXPERIENCE? WITH 0 BEING NOT AT ALL FRIGHTENING/DISTRESSING, AND 10 BEING VERY FRIGHTENING/DISTRESSING?** | | | |
| **B. FROM 0 TO 10, HOW IRRITATING OR FRUSTRATING WAS THIS EXPERIENCE? WITH 0 BEING NOT AT ALL IRRITATING/FRUSTRATING, AND 10 BEING VERY IRRITATING/FRUSTRATING?** | | | |
| **C. DID THIS EXPERIENCE MAKE YOU WORRY THAT YOU WERE LOSING YOUR MIND?**  Not at all (0) Somewhat (1) A lot (2) | | | |
| **D. DO YOU FIND YOUR CLOSE RELATIONSHIPS (E.G. WITH FAMILY) DIFFICULT BECAUSE OF THESE EXPERIENCES?**  Not at all (0) Somewhat (1) A lot (2) | | | |
| **E. ARE YOU ABLE TO IGNORE THESE EXPERIENCES?**  Not at all (2) Somewhat | (1) | A lot | (0) |
| **F. HAVE YOU STOPPED DOING THINGS YOU USED TO BECAUSE OF THESE EXPERIENCES?**  Not at all (0) Somewhat (1) A lot (2) | | | |

| **15. HAVE YOU EVER HEARD A VOICE OR SOUND WHEN NO ONE WAS THERE?**  No (0)  **GO TO NEXT SECTION**  Yes (1) **(STUDY PARTNER)** |
| --- |
| **PLEASE DESCRIBE WHAT YOU HAVE HEARD:** |
| **A. WHEN DID THIS FIRST START?** |

*If date unknown: 01/1900*

| M | M | / | Y | Y | Y | Y |
| --- | --- | --- | --- | --- | --- | --- |

1. **WHEN DID THIS LAST HAPPEN?**

*If date unknown: 01/1900*

| M | M | / | Y | Y | Y | Y |
| --- | --- | --- | --- | --- | --- | --- |

1. **APPROXIMATELY HOW LONG DO THESE EXPERIENCES USUALLY LAST?**

Seconds Minutes Hours

1. ***specify***
2. ***specify***
3. ***specify***

|  |
| --- |
|  |
|  |
|  |

Continuous while awake

1. **HOW OFTEN DO THEY USUALLY OCCUR?**

Less than every few months

Every few months Every few weeks Every few days Every few hours Every few minutes Every few seconds

Continuously- present throughout the day

(4)

(1)

(2)

(3)

(4)

(5)

(6)

(7)

(8)

1. **IN A TYPICAL MONTH, HOW MANY EXPERIENCES WOULD YOU HAVE?**

|  |  |  |  |  |
| --- | --- | --- | --- | --- |

1. **AND IN THIS TYPICAL MONTH, HOW MANY DAYS WOULD YOU HAVE THESE EXPERIENCES?**
2. **IN THE LAST 3 MONTHS, HOW MANY EXPERIENCES HAVE YOU HAD?**

|  |  |  |  |  |
| --- | --- | --- | --- | --- |

1. **AND IN THE LAST 3 MONTHS, HOW MANY DAYS WOULD YOU HAVE THESE EXPERIENCES?**
2. **IS THIS EXPERIENCE ASSOCIATED WITH FALLING ASLEEP OR WAKING UP?**

Never (0) Sometimes (1) Always (2)

| **J. AT WHAT TIME OF THE DAY DOES THIS EXPERIENCE USUALLY OCCUR?**  Night (0) Day time (1) Any time | (2) |
| --- | --- |
| **K. IS THIS EXPERIENCE ASSOCIATED WITH AN ODD SMELL OR TASTE?**  Never (0) Sometimes (1) Always | (2) |
| **L. DOES IT EVER FEEL LIKE IT IS TOUCHING YOU?**  Never (0) Sometimes (1) Always | (2) |
| **M. WHILST YOU ARE HAVING THE EXPERIENCE DO YOU EVER BELIEVE IT IS REAL?**  Never (0) Sometimes (1) Always | (2) |
| **N. DO YOU EVER ACT ON THE EXPERIENCE?**  Never (0) Sometimes (1) Always | (2) |
| **O. DO YOU HAVE AN EXPLANATION OF THESE EXPERIENCES THAT OTHERS SAY ARE NOT TRUE OR REAL?**  Never (0) Sometimes (1) Always (2) | |
